# Comparative safety of serotonin (5-HT_3_) receptor antagonists in patients undergoing surgery: a systematic review and network meta-analysis

**DOI:** 10.1186/s12916-015-0379-3

**Published:** 2015-06-18

**Authors:** Andrea C Tricco, Charlene Soobiah, Erik Blondal, Areti A Veroniki, Paul A Khan, Afshin Vafaei, John Ivory, Lisa Strifler, Huda Ashoor, Heather MacDonald, Emily Reynen, Reid Robson, Joanne Ho, Carmen Ng, Jesmin Antony, Kelly Mrklas, Brian Hutton, Brenda R Hemmelgarn, David Moher, Sharon E Straus

**Affiliations:** 1 grid.415502.7Li Ka Shing Knowledge Institute, St. Michael’s Hospital, 209 Victoria Street, East Building, Toronto, ON M5B 1W8 Canada; 2 grid.17063.33Epidemiology Division, Dalla Lana School of Public Health, University of Toronto, 6th floor, 155 College St, Toronto, ON M5T 3M7 Canada; 3 grid.17063.33Institute for Health Policy Management and Evaluation, University of Toronto, 4th Floor, 155 College St, Toronto, ON M5T 3M6 Canada; 4 grid.22072.350000000419367697Departments of Community Health Sciences, Faculty of Medicine, University of Calgary, TRW Building, 3rd Floor, 3280 Hospital Drive, Calgary, AB T2N 4Z6 Canada; 5 grid.412687.e0000000096065108Clinical Epidemiology Program, Centre for Practice-Changing Research, Ottawa Hospital Research Institute, 725 Parkdale Ave, Ottawa, ON K1Y 4E9 Canada; 6 grid.17063.33Department of Geriatric Medicine, University of Toronto, 27 King’s College Circle, Toronto, ON M5S 1A1 Canada

**Keywords:** Systematic review, Network meta-analysis, Serotonin receptor antagonists, Postoperative nausea, Postoperative vomiting

## Abstract

**Background:**

Serotonin (5-HT_3_) receptor antagonists are commonly used to decrease nausea and vomiting for surgery patients, but these agents may be harmful. We conducted a systematic review on the comparative safety of 5-HT_3_ receptor antagonists.

**Methods:**

Searches were done in MEDLINE, Embase, and the Cochrane Central Register of Controlled Trials to identify studies comparing 5-HT_3_ receptor antagonists with each other, placebo, and/or other antiemetic agents for patients undergoing surgical procedures. Screening search results, data abstraction, and risk of bias assessment were conducted by two reviewers independently. Random-effects pairwise meta-analysis and network meta-analysis (NMA) were conducted. PROSPERO registry number: CRD42013003564.

**Results:**

Overall, 120 studies and 27,787 patients were included after screening of 7,608 citations and 1,014 full-text articles. Significantly more patients receiving granisetron plus dexamethasone experienced an arrhythmia relative to placebo (odds ratio (OR) 2.96, 95 % confidence interval (CI) 1.11–7.94), ondansetron (OR 3.23, 95 % CI 1.17–8.95), dolasetron (OR 4.37, 95 % CI 1.51–12.62), tropisetron (OR 3.27, 95 % CI 1.02–10.43), and ondansetron plus dexamethasone (OR 5.75, 95 % CI 1.71–19.34) in a NMA including 31 randomized clinical trials (RCTs) and 6,623 patients of all ages. No statistically significant differences in delirium frequency were observed across all treatment comparisons in a NMA including 18 RCTs and 3,652 patients.

**Conclusion:**

Granisetron plus dexamethasone increases the risk of arrhythmia.

**Electronic supplementary material:**

The online version of this article (doi:10.1186/s12916-015-0379-3) contains supplementary material, which is available to authorized users.

## Background

Serotonin (5-HT_3_) receptor antagonists are a class of antiemetics recommended for patients undergoing surgery who are at risk for nausea and vomiting [1, 2]. Serotonin (5-HT_3_) receptor antagonists reduce nausea and vomiting by inhibiting vagal nerves in the central nervous system and intestinal mucosa [3]. However, some evidence suggests that 5-HT_3_ receptor antagonists can increase the risk of cardiac harm in children undergoing chemotherapy [4, 5]. Adverse events associated with these medications include a decrease in heart rate and prolongation of the QT interval. We were commissioned by Health Canada, a department of the federal government, to determine the comparative safety of 5-HT_3_ receptor antagonists for patients of all ages undergoing surgery due to safety concerns regarding the 5-HT_3_ receptor antagonists.

## Methods

We used an integrated knowledge translation approach [6], entailing collaboration between researchers and research users throughout the conduct of this study. The research users involved in this study who posed the original study question were from Health Canada.

### Protocol

A protocol was developed and revised using feedback from the research team and the research users. We registered our protocol with PROSPERO (CRD42013003564) and published it in an open-access journal [7]. Our methods are described briefly here; additional details can be found in the protocol publication. We originally intended to evaluate both safety and efficacy outcomes for patients undergoing surgery or chemotherapy; however, due to the enormous number of studies that met the inclusion criteria, we made slight changes to our protocol and subdivided the analyses. The current paper focuses on the safety of 5-HT_3_ antagonists in patients undergoing surgery. Subsequent papers will examine the efficacy of 5-HT_3_ antagonists for patients undergoing surgery [8], and the efficacy and safety of 5-HT_3_ antagonists for patients undergoing chemotherapy.

### Eligibility criteria

We included experimental (randomized clinical trials (RCTs), quasi-RCTs, non-RCTs), quasi-experimental (interrupted time series, controlled before–after studies), and observational (cohort) studies involving patients of any age undergoing any type of surgery who were given a 5-HT_3_ receptor antagonist for nausea and/or vomiting. A list of the agents and relevant comparators that were investigated in the included studies can be found in Additional file 1: Appendix 1. The primary outcome was the number of patients experiencing arrhythmia, and secondary outcomes were QT prolongation, PR prolongation, delirium, and mortality (overall and sudden cardiac death). Given the large number of included studies we limited the review to those published in English. Studies suspected or identified as fraudulent were excluded [9].

### Information sources

An experienced librarian executed searches of MEDLINE, Embase, and the Cochrane Central Register of Controlled Trials from inception until 11 January 2013. Unpublished studies were sought by searching trial protocol registries and conference proceedings.

### Study selection and data collection

After a calibration exercise, the literature search results were screened by pairs of reviewers, working independently. The same approach was used to abstract data and appraise the quality of included studies. Conflicts at both the screening and the abstraction steps were resolved through discussion. When data was missing or clarification of published data was needed we contacted the authors.

### Appraisal of methodological quality and risk of bias

To assess methodological quality and risk of bias of the included studies, we used the Cochrane Effective Practice and Organisation of Care risk of bias tool for experimental and quasi-experimental studies [10], the Newcastle-Ottawa Scale [11] for cohort studies, and the McMaster Quality Assessment Scale of Harms (known as the McHarm tool) [12] for studies reporting harms.

### Synthesis of included studies

A pooled estimate of effect was derived on the odds ratio (OR) scale using random-effects pairwise meta-analysis for each outcome and comparison, if at least two studies were available. When studies reported zero events in one treatment arm, 0.5 was added to the numerator and 1 was added to the denominator. Studies with zero events in both arms were excluded from the analyses. Between-study heterogeneity for direct-comparison meta-analysis was estimated using the restricted maximum likelihood (REML) [13] and measured using the *I*^*2*^ statistic [14]. Each pairwise meta-analysis estimate is presented along with the corresponding 95% confidence interval (CI). These analyses were conducted using the metafor package [15] in R 3.1.2 [16].

Before embarking on network meta-analysis (NMA), we evaluated the transitivity assumption by examining the comparability of the distributions of age (children versus adults), timing of administration (all time points versus during surgery), and risk of bias (all versus removing high risk of bias for randomization, allocation concealment, and blinding of outcome assessor) as potential treatment-effect modifiers across comparisons [17]. For each outcome, we visually inspected the potential effect modifiers by using colored edges in the network according to the level of the effect modifier and the majority of trials included in each comparison [18]. We evaluated the consistency assumption for the entire network using the design-by-treatment interaction model [19]. In case we found statistically significant inconsistency, we planned to assess certain paths of the network using the loop-specific method [20, 21] to identify which piece of evidence was responsible for the inconsistency (i.e., local inconsistency). We also planned to apply network meta-regression to adjust for potential effect modifiers if local inconsistency was identified. NMAs were performed within a frequentist framework, assuming a common within-network estimate for the heterogeneity parameter across all comparisons and estimated with the REML [13, 19]. We used the surface under the cumulative ranking (SUCRA) curve to rank the safety of the various 5-HT_3_ receptor antagonists [22].

The treatment nodes were selected with input from clinicians, pharmacists, and statisticians on the team. Due to the complexity of the analysis, we did not account for differences in doses and durations assuming that all impact the treatment effect equally. Specifically, when a study compared different doses of an intervention against another intervention, we included only the recommended dose in the analysis [1, 23–33].

The summary treatment effect generated by each NMA is presented along with its 95 % CI and 95 % predictive interval (PrI). The PrI, representing the interval within which the estimated treatment effect of a future study is expected to lie, captures the uncertainty of the NMA estimate and the magnitude of heterogeneity within the network overall [34, 35]. To assess the presence of reporting bias (including publication bias and small-study effects), we applied the comparison-adjusted funnel plot for each outcome separately [18]. We ordered the treatments from oldest to newest and then plotted the difference between each study-specific treatment effect and the corresponding comparison-specific summary effect under the fixed-effect model, against the study-specific standard error. We carried out subgroup analyses for all outcomes according to the timing of administration of 5-HT_3_ receptor antagonist therapy (all time periods versus during surgery) and age (all ages versus children). To establish the robustness of our results, we performed a sensitivity analysis in which we excluded studies with high risk of bias because of incomplete outcome data. Given that our primary analysis was a network meta-analysis restricted to RCTs, we conducted a second sensitivity analysis in which non-randomized studies were added to the network, to observe the contribution of different study designs to the treatment effects. Network meta-analyses were conducted using the mvmeta command in Stata 13.0 [36, 37].

## Results

### Literature search

**Fig. 1:**
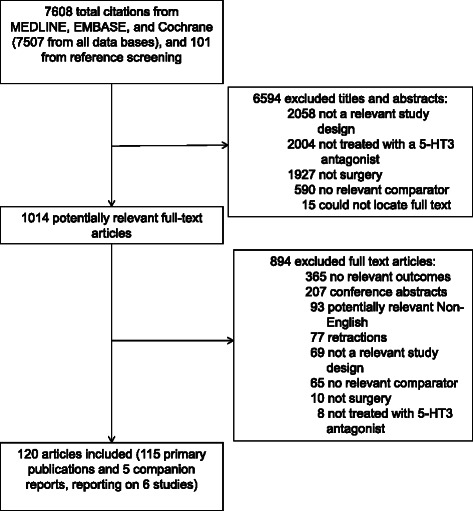
Study flow. Details the flow of information through the different phases of the review; maps out the number of records identified, included and excluded, and the reasons for their exclusion

After screening 7,608 citations, we reviewed 1,014 potentially relevant full-text articles and identified 115 primary publications [10, 33, 38–150] and five companion reports [151–154] (reporting on six studies) providing data on 27,787 patients that met our inclusion criteria (Fig. 1). Overall, 77 studies were excluded because they reported results suspected or confirmed to be fraudulent [9]. One of the included studies was an unpublished conference abstract [84].

### Study and patient characteristics

The majority of the included studies were RCTs (97 %), conducted in Europe (37 %), North America (26 %), or Asia (24 %) and published between 1990 and 2013 (Table 1, Additional file 1: Appendix 2). The duration of follow-up was very short, ranging from ≤6 h to more than a week. The most frequent follow-up time observed was 12 to 24 h (69 %). The setting was not reported in the majority of trials (62 %) (Table 1).

**Table 1:** Study characteristics

Characteristic	Number of studies^a^ (n = 115)	Percentage of studies (%)
**Year of publication**		
1990–1994	7	6.1
1995–1999	37	32.2
2000–2004	19	16.5
2005–2009	36	31.3
2010–2013	16	13.9
**Geographic region**		
Europe	42	36.5
North America	30	26.1
Asia	28	24.3
Multi-continent	8	7.0
Australasia	3	2.6
Africa	2	1.7
Not reported	1	0.9
South America	1	0.9
**Study design**		
Randomized clinical trial	112	97.4
Non-randomized clinical trial	2	1.7
Controlled before–after study	1	0.9
**Study conduct period**		
1990–1999	1	0.9
2000–2009	15	13.0
2010–2013	1	0.9
Not reported	98	85.2
**Duration of follow-up** ^**b**^		
0 to ≤6	9	7.8
>6 to ≤12	2	1.7
>12 to ≤24	79	68.7
>24 to ≤48	17	14.8
>48 to ≤72	2	1.7
>72 to ≤1 week	3	2.6
Not reported	3	2.6
**Interventions examined: frequency** ^**c**^		
*Serotonin antagonists:* Reported as administered alone (administered with dexamethasone)		
Ondansetron	79 (7)	68.70 (6.1)
Granisetron	14 (4)	12.2 (3.5)
Tropisetron	15 (0)	13.0 (0.0)
Dolasetron	15 (1)	13.0 (0.9)
Palonosetron	4 (0)	3.5 (0.0)
Ramosetron	3 (1)	2.6 (0.9)
*Comparator antiemetics:*		
Butyrophenone	26	22.61
Benzamide	14	12.17
Dexamethasone	6	5.2
Phenothiazine	2	1.7
Antihistamine	3	2.61
NK-1	4	3.5
Anticholinergic	0	0
*Serotonin antagonists given with other antiemetic:*		
Serotonin antagonist + dexamethasone	13	11.3
Serotonin antagonist + butyrophenone	5	4.4
Serotonin antagonist + benzamide	0	0
Serotonin antagonist + antihistamine	1	0.9
Serotonin antagonist + NK-1	1	0.9
Serotonin antagonist + phenothiazine	0	0
*Placebo or no treatment*	86	74.78
**Outcomes examined: frequency** ^d^		
Arrhythmia	53	46.1
Delirium	34	29.6
Mortality	28	24.3
QT prolongation	18	15.7
**Setting**		
Not reported	71	61.7
Hospital	25	21.7
Medical center	16	13.9
Multi-center	3	2.6

^a^Includes unpublished data [84]; ^b^duration is in hours unless otherwise noted; ^c^multiple interventions and comparators examined across the studies; ^d^multiple interventions and outcomes reported per study. NK-1: Neurokinin 1 receptor antagonist

The interventions examined were ondansetron (0.1−48 mg/day) (69 %), granisetron (0.1−3 mg/day) (12 %), tropisetron (0.3−5 mg/day) (13 %), dolasetron (12.5−200 mg/day) (13 %), palonosetron (0.025−0.07 mg/day) (4 %), and ramosetron (0.1−0.6 mg/day) (3 %). Some studies examined 5-HT_3_ receptor antagonists administered concomitantly with other antiemetics, dexamethasone (2–16 mg/day) (11 %) and droperidol (2.5 mg/day) (4 %), being the most common (Table 1, Additional file 1: Appendix 3).

Arrhythmia was the most frequently reported outcome (46 %). Only five studies reported QT prolongation, and 13 reported on the QT interval. None of the studies reported the number of patients experiencing PR prolongation or sudden cardiac death. We abstracted data from all of the included studies, and included 51 studies in our analyses. Reasons for excluding studies from the analyses included the manner in which the outcome was reported (e.g., mean versus number of patients), reporting zero events for all treatment arms, and investigating a single 5-HT_3_ receptor antagonist (with a different dosage in each treatment arm).

The average sample size was 242 participants ranging from 28 to 1,044, and 64% of participants were women (Table 2, Additional file 1: Appendix 4). Most of the studies involved only adult patients (63 %), patients with American Society of Anesthesiologists physical status I or II (58 %), and patients who were undergoing obstetrical and gynecological (32 %) surgery. Patients’ history of postoperative nausea and vomiting was reported in 58% of the studies, and history of motion sickness was reported in 43 % of the studies. Comorbidities were rarely reported (6 %) (Table 2).

**Table 2:** Patient characteristics

Total number of patients	27,787	
Mean sample size	242	
Mean percentage female (%)	64	
	Number of studies (n = 115)^a^	Percentage of studies (%)
**Age category**		
Children only (aged <18 years)	22	19.1
Adults only (aged ≥18 years to ≤65 years)	72	62.6
Children and adults (aged ≤65 years)	2	1.7
Adults and elderly (aged ≥18 years)	16	13.9
All ages	2	1.7
Not reported	1	0.9
**American Society of Anesthesiologists (ASA) physical status**		
I	4	3.5
I or II	62	53.9
I or II or III	32	27.8
Not reported	17	14.8
**Surgery type**		
Obstetric and gynecological	37	32.2
Eye	12	10.4
Gastrointestinal	9	7.8
General dentistry, oral and maxillofacial surgery, and orthodontics	5	4.3
Orthopedic	5	4.3
Neurological	3	2.6
Otolaryngological	2	1.7
Breast	1	0.9
Cardiovascular	1	0.9
Urological	1	0.9
Miscellaneous (includes multiple surgery types, abdominal surgery, and plastic surgery unspecified)	39	33.9
**History of motion sickness**		
Yes	49	42.6
No or not reported	66	57.4
**History of postoperative nausea and vomiting**		
Yes	67	58.3
No or not reported	48	41.7
**Comorbidities** ^**b**^		
Not reported	109	94.8
Diabetes mellitus	3	2.6
Cardiovascular	2	1.7
Obesity	1	0.9
Urological	1	0.9
Migraines	1	0.9
Liver disease	1	0.9

^a^Includes unpublished data; ^b^some studies considered more than one comorbidity

### Methodological quality and risk of bias

The majority of the included experimental and quasi-experimental studies had unclear or high risk of bias on the following items: allocation concealment (57 %), similarity of baseline outcome characteristics (88 %), incomplete outcome data (51 %), selective outcome reporting bias (97 %), and other types of bias, including the potential for funding bias because the study was funded by private industry and an author on the publication was employed by the company sponsoring the study (88 %) (Additional file 1: Appendix 5, 6). None of the 115 studies reporting harms outcomes fully reported all items on the McHarm tool (Additional file 1: Appendix 7, 8). The visual inspection of the comparison adjusted funnel plots showed that there was no evidence for small-study effects and publication bias (Additional file 1: Appendix 9).

### Statistical analysis

#### Arrhythmia

**Fig. 2:**
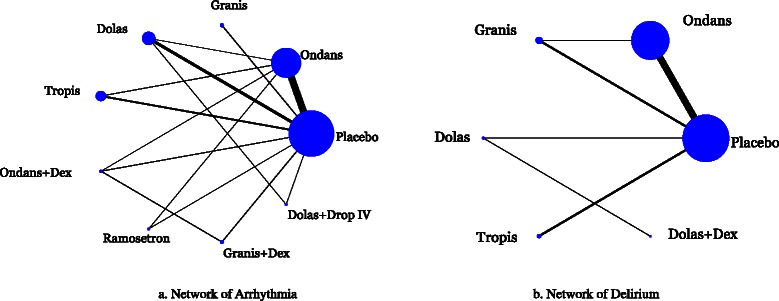
Network meta-analysis diagrams for (**a**) arrhythmia and (**b**) delirium. Nodes are proportional to the number of patients included in the corresponding treatments, and edges are weighted according to the number of studies included in the respective comparisons. Dex: Dexamethasone; Dolas: Dolasetron; Drop: Droperidol; Granis: Granisetron; Ondans: Ondansetron; Tropis: Tropisetron

**Fig. 3:**
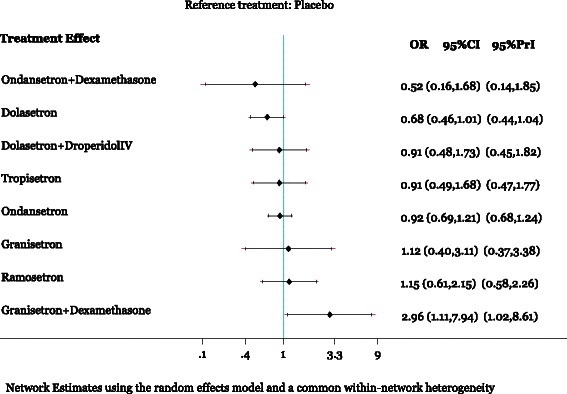
Network meta-analysis results for arrhythmia. All treatments are compared to placebo. The black horizontal lines represent the 95 % confidence intervals (CI) of the summary treatment effects and red horizontal lines the 95 % predictive intervals (PrI). Results are presented on the odds ratio (OR) scale. Among patients of all ages receiving granisetron plus dexamethasone, significantly more experienced arrhythmia compared with placebo (OR 2.96, 95 % CI 1.11–7.94), ondansetron (OR 3.23, 95 % CI 1.17–8.95), dolasetron (OR 4.37, 95 % CI 1.51–12.62), tropisetron (OR 3.27, 95 % CI 1.02–10.43), and ondansetron plus dexamethasone (OR 5.75, 95 % CI 1.71–19.34). Note: Reference treatment is placebo. CI: Confidence interval; OR: Odds ratio; PrI: Predictive interval

The network meta-analysis for arrhythmia included 31 RCTs with 6,623 patients [40, 43, 45, 53, 59, 74, 78, 79, 83, 86–89, 97, 102, 108, 112–115, 117, 119, 123, 125, 128, 130, 132, 138, 141, 142, 150]. The network geometry and included drugs can be found in Fig. 2a. Twenty-one studies were excluded from the analysis because they reported zero events in all arms [39, 43, 57, 60, 61, 71, 73, 81, 82, 86, 90, 92–94, 98, 110, 121, 127, 145, 155, 156]. Although the definitions of arrhythmia varied across the studies (Additional file 1: Appendix 10), there was no evidence of network inconsistency (*χ*^*2*^ = 3.49, degrees of freedom = 10, *P* = 0.968, heterogeneity variance = 0.01), and the within-network heterogeneity variance was estimated to be 0.00. Among patients of all ages receiving granisetron plus dexamethasone, significantly more experienced arrhythmia compared with placebo (OR 2.96, 95 % CI 1.11–7.94), ondansetron (OR 3.23, 95 % CI 1.17–8.95), dolasetron (OR 4.37, 95 % CI 1.51–12.62), tropisetron (OR 3.27, 95 % CI 1.02–10.43), and ondansetron plus dexamethasone (OR 5.75, 95 % CI 1.71–19.34) (Fig. 3, Table 3, Additional file 1: Appendix 11). According to the SUCRA curves (Additional file 1: Appendix 12), the safest agents for arrhythmia were ondansetron plus dexamethasone (83 % probability) and dolasetron (82 % probability).

**Table 3:** Statistically significant results of network meta-analysis for all time periods of drug administration

	All ages			Children only		
Treatment comparison	Number of studies	MA estimate: OR (95 % CI)	NMA estimate: OR (95 % CI)	Number of studies	MA estimate: OR (95 % CI)	NMA estimate: OR (95 % CI)
Arrhythmia	31 RCTs and 6,623 patients			9 RCTs and 1,572 patients		
Granisetron + DEX vs placebo	2	2.63 (0.75– 9.29)	2.96 (1.11–7.94)	1	4.89 (1.15–20.79)	5.15 (1.33–19.91)
Granisetron + DEX vs ondansetron	NA	NA	3.23 (1.17–8.95)	NA	NA	4.71 (1.08–20.46)
Granisetron + DEX vs dolasetron	NA	NA	4.37 (1.51–12.62)	NA	NA	NA
Granisetron + DEX vs tropisetron	NA	NA	3.27 (1.02–10.43)	NA	NA	NA
Granisetron + DEX vs ondansetron + DEX	2	8.10 (1.92–34.13)	5.75 (1.71–19.34)	1	7.67 (1.47–40.00)	7.12 (1.66–30.63)

CI: Confidence interval; DEX: Dexamethasone; MA: Meta-analysis; NA: Not applicable; NMA: Network meta-analysis; OR: Odds ratio

A subgroup analysis was conducted for 26 RCTs involving 4,878 patients in which the agents were administered during surgery [40, 43, 45, 53, 59, 74, 78, 79, 83, 86–89, 97, 102, 112, 113, 115, 117, 119, 123, 125, 132, 138, 142, 150]. The results were the same as for the primary analysis, except that significantly fewer patients of all ages receiving dolasetron experienced arrhythmia compared with placebo (OR 0.58, 95 % CI 0.36–0.93) and ramosetron (OR 0.38, 95 % CI 0.17–0.92) (Additional file 1: Appendix 13). According to the SUCRA curves for this subgroup analysis, the safest agents were dolasetron (86 % probability) and ondansetron plus dexamethasone (83 %).

Another subgroup analysis was conducted for nine RCTs involving a total of 1,572 patients to examine the intra-operative administration of ondansetron, ondansetron plus dexamethasone, and granisetron plus dexamethasone to children (Table 3, Additional file 1: Appendix 13) [53, 79, 86, 89, 97, 113, 117, 123, 138]. Significantly more children receiving granisetron plus dexamethasone during surgery experienced arrhythmia compared with placebo (OR 5.15, 95 % CI 1.33–19.91), ondansetron (OR 4.71, 95 % CI 1.08–20.46), and ondansetron plus dexamethasone (OR 7.12, 95 % CI 1.66–30.63). According to the SUCRA curves, the safest agent in terms of arrhythmia was ondansetron plus dexamethasone (80 % probability). Finally, a sensitivity analysis was conducted in which one RCT was removed because of high risk of incomplete outcome data [128], and the same results were observed (Additional file 1: Appendix 13).

#### Delirium

The network meta-analysis for delirium included 18 studies involving 3,652 patients in which ondansetron, granisetron, dolasetron, tropisetron, and dolasetron plus dexamethasone were administered during surgery [52, 60, 68, 69, 76, 79, 96, 100, 105, 106, 118, 124, 128, 133, 137, 139, 144, 146]. The network geometry and included drugs can be found in Fig. 2b. Ten studies were excluded from the analysis because they reported zero events in all arms [49, 69, 75, 90, 99, 103, 129, 135, 140, 143]. No statistically significant results were observed and the within-network heterogeneity variance in the network meta-analysis model was estimated to be 0.00 (Additional file 1: Appendix 14). Although the definitions of delirium varied across the studies (Additional file 1: Appendix 15, 16), there was no evidence of network inconsistency (*χ*^*2*^ = 0.32, degrees of freedom = 2, *P* = 0.851, heterogeneity variance = 0.00).

#### Mortality

A meta-analysis was conducted for three studies including 1,255 patients that reported mortality for comparisons of ondansetron with placebo [10, 111, 142]. No statistically significant effects were observed (OR 1.92, 95 % CI 0.30–12.21). Twenty-five studies were excluded from this analysis because they reported zero events in both arms [38, 41, 44, 55, 56, 58, 62, 67, 70, 72, 77, 78, 80, 107, 109, 115, 120, 126, 128, 130, 131, 134, 149, 157, 158].

#### QT prolongation

Two RCTs reported the number of patients experiencing QT prolongation [55, 116]. In one of these studies, there was no statistically significant difference between ondansetron and placebo (OR 0.75, 95 % CI 0.47–1.20) [55], and in the other there was no statistically significant difference between granisetron and placebo (OR 0.32, 95 % CI 0.01–8.02) [116]. Three studies did not inform the analysis and were excluded, as they reported zero events in both arms [58, 115, 159].

## Discussion

More patients receiving granisetron plus dexamethasone experienced arrhythmia compared to other agents. The safest 5-HT_3_ receptor antagonists with respect to arrhythmia were ondansetron plus dexamethasone and dolasetron for patients of all ages and ondansetron plus dexamethasone for children (none of the included studies examined dolasetron in children). These results were consistent across subgroup and sensitivity analyses. None of the agents caused significantly more patients to experience delirium. Few studies reported QT prolongation, and no statistically significant results for this outcome were reported in the two studies reporting at least one event. As well, no statistically significant differences in mortality were observed between ondansetron and placebo in a meta-analysis of three studies that reported this outcome. None of the studies included in this analysis reported the number of patients experiencing PR prolongation or sudden cardiac death.

Our finding of no increased risk of cardiac arrhythmia in association with ondansetron therapy supports the results of a previous systematic review [160]. Although we are aware of other systematic reviews and meta-analyses of 5-HT_3_ receptor antagonists [9, 161], the previous researchers did not conduct network meta-analysis, so we cannot compare our results with theirs. Notably, because of our comprehensive literature search and broad eligibility criteria, we included 62 studies involving a total of 14,705 patients that were not included in any of the previous reviews (Additional file 1: Appendix 17).

We found no increased risk of arrhythmia with dolasetron for patients of any age. This does not mean that a cardiac risk does not exist; we found no studies examining other cardiac harms, such as PR prolongation and sudden cardiac death. We identified no studies examining dolasetron administered to children. We found other data gaps through the conduct of this review. In particular, most of the studies focused on effectiveness outcomes, and relatively few reported harms. Our network meta-analysis results for the effectiveness outcomes have been reported in another publication [8].

The studies included in our analysis had some methodological limitations. Most of the studies were small (average sample size 242 patients) and larger sample sizes are required to assess harms, in particular harms that occur only rarely, such as arrhythmia and delirium. Indeed, the need for larger sample sizes is the reason we included non-randomized studies in our review. Although these non-randomized studies involved more patients than the RCTs, their inclusion did not change the network meta-analysis results obtained for arrhythmia or delirium. As well, many of the studies failed to report baseline characteristics or all items assessed by the McHarm tool, and many of the included trials had an unclear or high risk of bias on important items for the conduct of trials, including allocation concealment, selective outcome reporting bias, and potential for funding bias.

Our systematic review process also had some limitations. Slight changes to our original protocol [7] were necessary, because of the enormous number of studies that met our inclusion criteria. For example, we were unable to report data on patients undergoing chemotherapy in this paper (but these will be disseminated in an upcoming paper), we did not include studies written in languages other than English, and we focused inclusion to unpublished conference abstracts from the past 10 years that included relevant data. However, we were able to include unpublished data from one study [84], and our funnel plots showed no evidence of small-study effects or publication bias. Furthermore, we assumed that the effects of the different doses and durations were identical across the treatments, and that they defined the same node they belong to. We are currently exploring these assumptions in another paper [162]. Finally, we had to exclude 77 studies because they contained data known or suspected to be fraudulent, as identified by editors and authors in the field and presented in a paper [9]; we did not conduct a sensitivity analysis including these articles to examine the effect of excluding these studies on our results.

## Conclusion

We conclude that most 5-HT_3_ receptor antagonists that do not cause delirium. Granisetron plus dexamethasone increased the risk of cardiac harm (arrhythmia), with the number needed to harm ranging from five to eight. We are unable to comment on the relationship between 5-HT_3_ receptor antagonists and other cardiac harms, such as for PR prolongation and sudden cardiac death, as no studies reported these important outcomes.

## Additional file

Additional file 1:
**Appendix 1–17. Includes 17 appendices with supplementary data.**

## Abbreviations

5-HT_3_: Serotonin
CI: Confidence interval
IV: Intravenous
NK-1: Neurokinin 1 receptor antagonist
NMA: Network meta-analysis
OR: Odds ratio
PrI: Predictive interval
RCT: Randomized control trial
REML: Restricted maximum likelihood
SUCRA: Surface under the cumulative ranking
