# Supplementary material for: Comparative safety of serotonin (5-HT3) receptor antagonists in patients undergoing surgery: a systematic review and network meta-analysis
Source: BMC Med. 2015 Jun 18;13:142. doi: 10.1186/s12916-015-0379-3 (PMC4472408; doi:10.1186/s12916-015-0379-3)
Supplement: Additional file 1: — Appendix 1–17. Includes 17 appendices with supplementary data. [file 12916_2015_379_MOESM1_ESM.pdf]

# APPENDICES 1-17

**Appendix 1. Inclusion criteria for interventions and comparator groups ............................................ 2**

**Appendix 2. Study characteristics ..................................................................................................... 3**

**Appendix 3. Frequencies for specific drugs and drug combinations ..................................................... 8**

**Appendix 4. Patient characteristics.................................................................................................... 9**

**Appendix 5. Cochrane EPOC Risk-of-bias appraisal results ..................................................................13**

**Appendix 6. Aggregate EPOC risk-of-bias appraisal results ...................................................................16**

**Appendix 7. McHarm appraisal results....................................................................................................17**

**Appendix 8. Aggregate McHarm appraisal results ..................................................................................19**

**Appendix 9 Comparison adjusted funnel plots.........................................................................................20**

**Appendix 10. Definitions of arrhythmia ..................................................................................................21**

**Appendix 11. Network meta-analysis for all outcomes for all time periods ..........................................22**

**Appendix 12. Plot of the surface under the cumulative ranking (SUCRA) curves for the 9 treatments included in Arrhythmia. ..................................................................................................................24**

**Appendix 13: Subgroup analysis for Arrhythmia and Delirium...........................................................25**

**Appendix 14. Network meta-analysis results for Delirium. ....................................................................28**

**Appendix 15. Definitions of delirium .......................................................................................................29**

**Appendix 16. Plot of the surface under the cumulative ranking (SUCRA) curves for the 6 treatments included in Delirium. .........................................................................................................................30**

**Appendix 17. Included studies in our review versus previous systematic reviews ...............................31**

## Appendix 1. Inclusion criteria for interventions and comparator groups

| *Intervention:* | *Eligible comparators:* |
| --- | --- |
| 5-HT3 receptor antagonist with or without dexamethasone (Ondansetron, Granisetron, Dolasetron, Tropisetron, Ramosetron, Palonosetron) | I. Different 5-HT3 antagonist with or without Dexamethasone |
| | II. Antiemetic comparator listed below with or without Dexamethasone |
| | III. Same 5-HT3 antagonist given at different dose, formula, and/or timing or same 5-ht3 with Dexamethasone |
| | IV. Same 5-HT3 antagonist given in combination with one of the comparators below |
| | V. Different 5-HT3 antagonist given in combination with one of the comparators below |

*Antiemetic comparators included:*

| Benzamides | Phenothiazine | Butyrophenones | Antihistamines | NK-1 | Steroids |
| --- | --- | --- | --- | --- | --- |
| Metoclopramide | Perphenazine | Haloperidol | Dimenhydrinate | Aprepitant | Dexamethasone |
| | Promethazine | Droperidol | Cyclizine | Casopitant | |
| | | | | Rolapitant | |

**Abbreviations**: 5-HT3, Serotonin; NK-, Neurokinin-1 receptor antagonists; NaSSA, Noradrenergic and specific serotonergic antidepressant.

## Appendix 2. Study characteristics

| Author, year | Country of conduct | Study conduct period | Study duration (hours) | Setting | Interventions examined | Outcomes Reported |
|---|---|---|---|---|---|---|
| ***Randomized clinical trials (n=112)*** | | | | | | |
| Choi, 2012 | South Korea | NR | 48 | NR | Ondansetron + Dexamethasone, Ramosetron + Dexamethasone | Mortality |
| Wang, 2012 | China (Taiwan) | NR | 24 | NR | Dexamethasone, Haloperidol + Dexamethasone, Ondansetron + Dexamethasone | QT Interval, Arrhythmia |
| Ekinci, 2011 | Turkey | February 2007 - March 2008 | 48 | NR | Droperidol, Metoclopramide, Ondansetron, Placebo, Tropisetron | Arrhythmia |
| El-deeb, 2011b | Egypt | NR | 24 | NR | Granisetron + Dexamethasone, Placebo | Arrhythmia |
| Gan, 2011 | USA, Canada | NR | 119 | NR | Ondansetron, Placebo, Rolapitant | Mortality |
| Gupta, 2011 | India | October 2008 - April 2009 | NR | Hospital | Ondansetron, Placebo | QT Interval |
| Park, 2011 | Republic of Korea | June 2010 - September 2010 | 24 | Hospital | Ondansetron, Palonosetron | Mortality |
| Ryu, 2011 | South Korea | NR | 48 | NR | Ramosetron, Tropisetron | QT Interval |
| Sahoo, 2011 | India | September 2008 - December 2008 | 1 | NR | Ondansetron, Placebo | Arrhythmia |
| Bilgin, 2010 | Turkey | NR | 24 | NR | Dexamethasone, Metoclopramide, Ondansetron, Placebo | Mortality |
| Choi, 2010 | Korea | September 2008 - February 2009 | 48 | NR | Ondansetron, Placebo, Ramosetron | Arrhythmia |
| Jee, 2010 | Korea | NR | 36 | NR | Metoclopramide + Dexamethasone, Ondansetron + Dexamethasone | Delirium, Arrhythmia |
| Jokela, 2010 | Finland | December 2007 - January 2009 | 24 | Hospital | Ondansetron, Placebo | Delirium |
| Mehta, 2010 | Canada | February 2008 - March 2009 | 2 | NR | Droperidol, Ondansetron, Ondansetron + Droperidol, Placebo | QT Interval |
| Shakya, 2010 | India | NR | 2 | NR | Ondansetron, Placebo | Delirium |
| Singla, 2010 | Belgium, Canada, Germany, Hungary, Spain, USA. | NR | 119 | Multi-centre | Casopitant, Ondansetron, Ondansetron + Casopitant | Mortality |
| Feng, 2009 | Taiwan | NR | 24 | NR | Haloperidol, Ondansetron, Ondansetron + Haloperidol | QT Interval |
| Jain, 2009 | India | NR | 24 | NR | Granisetron, Ondansetron, Placebo | Delirium |

| | | | | | | |
|---|---|---|---|---|---|---|
| Riad, 2009 | Saudi Arabia | NR | 24 | NR | Granisetron + Dexamethasone, Ondansetron + Dexamethasone, Placebo | Arrhythmia |
| Rosow, 2009 | USA | NR | NR | Hospital | Haloperidol, Ondansetron | Delirium, QT Interval |
| Candiotti, 2008 | USA, Romainia | NR | 72 | NR | Palonosetron, Placebo | Mortality, QT Interval |
| Choi, 2008 | South Korea | September 2006 - June 2007 | 48 | NR | Ondansetron, Ramosetron | Mortality |
| Contreras-Dominguez, 2008 | Chile | December 2003 - February 2006 | 48 | Hospital | Dexamethasone, Droperidol, Granisetron, Metoclopramide, Placebo, Tropisetron | Arrhythmia |
| Kovac, 2008 | Germany, Poland, Czech Republic | NR | 72 | Multi-centre | Palonosetron, Placebo | Mortality, QT Interval |
| Owczuk, 2008 | Poland | NR | 0 | NR | Ondansetron, Placebo | Arrhythmia |
| Piper, 2008 | Germany, Switzerland | 1999 - 2004 | 24 | NR | Dolsetron, Droperidol, Placebo | Delirium, Arrhythmia |
| Said-Ahmed, 2008 | Egypt | NR | 24 | NR | Granisetron, Placebo | Arrhythmia |
| Bestas, 2007 | Turkey | NR | 24 | Medical center | Granisetron, Ondansetron, Placebo | Mortality |
| Diemunsch, 2007 | North America (including USA), South America, Europe, Australia, Asia | May 2004 - April 2005 | 48 | NR | Aprepitant, Ondansetron | QT Interval, Arrhythmia |
| Gan, 2007 | Multicentred | September 2003 - November 2004 | 48 | NR | Aprepitant, Ondansetron | QT Interval |
| Han, 2007 | Korea | NR | 48 | NR | Ondansetron | Arrhythmia |
| Lee, 2007 | Republic of China (Taiwan) | NR | 24 | NR | Haloperidol, Ondansetron | QT Interval, Arrhythmia |
| Oksuz, 2007 | Turkey | NR | 24 | NR | Granisetron, Metoclopramide, Ondansetron | Mortality |
| Rusch, 2007 | Germany | NR | 24 | Hospital | Dolsetron, Dolsetron + Dexamethasone, Haloperidol, Haloperidol + Dexamethasone | Delirium |
| Sagir, 2007 | Turkey | NR | 0.25 | NR | Granisetron, Placebo | Delirium |
| Sandhu, 2007 | Thailand | NR | 24 | NR | Metoclopramide, Ondansetron | Mortality |
| Siddik-Sayyid, 2007 | Lebanon | NR | 24 | Medical center | Granisetron, Ondansetron, Placebo | Arrhythmia |
| Bridge, 2006 | USA | NR | 24 | Hospital | Dolsetron, Granisetron, Ondansetron | Mortality |
| Chan, 2006 | USA, Hong Kong, New Zeland | September 2002 - June 2004 | 48 | NR | Droperidol, Ondansetron, Ondansetron + Droperidol, Placebo | Arrhythmia |
| Kelsaka, 2006 | Turkey | NR | NR | NR | Ondansetron, Placebo | Arrhythmia |
| Sarvela, 2006 | Finland | January 2003 - November 2003 | 24 | NR | Ondansetron, Placebo, Tropisetron | Delirium |
| Tosun, 2006 | Turkey | NR | 24 | NR | Placebo, Tropisetron | Delirium |
| D'Angelo, 2005 | USA | March 2002 - August 2002 | 24 | Multi-centre | Granisetron, Placebo | Mortality |
| Gan, 2005 | USA | NR | 24 | NR | Granisetron + Dexamethasone, | Mortality, Arrhythmia |

| | | | | | | |
|---|---|---|---|---|---|---|
| | | | | | Ondansetron + Dexamethasone | |
| Khalil, 2005 | USA, Canada | NR | 24 | Multi-centre | Ondansetron, Placebo | Delirium, Arrhythmia |
| Kocamanoglu, 2005 | Turkey | NR | 24 | NR | Droperidol, Granisetron, Granisetron + Dexamethasone, Granisetron + Droperidol, Placebo | Mortality |
| Kontrimaviciute, 2005 | Lithuania | NR | 24 | Hospital | Ondansetron, Placebo | Arrhythmia |
| Pirat, 2005 | Turkey | NR | 24 | NR | Ondansetron, Placebo | Arrhythmia |
| Treschan, 2005 | Germany | NR | 24 | NR | Ondansetron, Placebo | Arrhythmia |
| White, 2005[†] | USA | NR | 24 | NR | Palonosetron, Placebo | QT Interval |
| Apfel, 2004 (CR: Jokela, 2009) | Germany, USA, Finland, UK, Turkey, Slovakia | February 2000 - July 2002 | 24 | NR | Dexamethasone, Droperidol, Droperidol + Dexamethasone, Ondansetron, Ondansetron + Dexamethasone, Ondansetron + Droperidol | QT Interval |
| Binstock, 2004 | USA | NR | 24 | NR | Ondansetron, Placebo | Arrhythmia |
| Eberhart, 2004 | Germany | NR | 24 | NR | Dolsetron, Dolsetron + Droperidol, Droperidol, Placebo | Arrhythmia |
| Hanaoka, 2004 | Japan | NR | 24 | Multi-centre | Granisetron, Placebo | Arrhythmia |
| Samakarndi, 2004 | Saudi Arabia | NR | 24 | NR | Dexamethasone, Ondansetron, Ondansetron + Dexamethasone, Placebo | Arrhythmia |
| Charuluxananan, 2003 | Thailand | NR | 24 | Hospital | Ondansetron, Placebo | Arrhythmia |
| Loewen, 2003 | Canada | May 2000 - December 2001 | 24 | NR | Dolsetron, Droperidol | Delirium |
| O'Brien, 2003 | UK | NR | 24 | Hospital | Cyclizine, Ondansetron, Placebo | Arrhythmia |
| Argiriadou, 2002 | Greece | NR | 12 | Hospital | Ondansetron, Placebo, Tropisetron | Arrhythmia |
| Gurkan, 2002 | Turkey | NR | 6 | NR | Ondansetron, Placebo | Arrhythmia |
| Dabbous, 2001 | Lebanon | NR | 24 | NR | Droperidol, Metoclopramide, Ondansetron | Delirium |
| Kathirvel, 2001 | India | NR | 24 | NR | Ondansetron, Placebo | Delirium |
| Subramaniam, 2001 | India | NR | 24 | NR | Dexamethasone, Ondansetron, Placebo | Arrhythmia |
| Ahmed, 2000 | UK | NR | 24 | NR | Ondansetron, Ondansetron + Cyclizine, Placebo | Arrhythmia |
| Charuluxananan, 2000 | Thailand | NR | 4 | Hospital | Ondansetron, Placebo | Delirium |
| Jensen, 2000 | australia | NR | 24 | Hospital | Placebo, Tropisetron | Delirium |
| Kreisler, 2000 | USA | NR | 24 | NR | Droperidol, Ondansetron, Promethazine | Delirium |
| Philip, 2000 | USA | NR | 24 | Multi-centre | Dolsetron, Placebo | Arrhythmia |
| Zarate, 2000 | USA | NR | 24 | NR | Dolsetron, Ondansetron | Delirium |
| Koivuranta, 1999 | Finland | NR | 24 | Hospital | Ondansetron, Tropisetron | Delirium |
| McCall, 1999 | USA | NR | 8 | NR | Dimenhydrinate, Ondansetron, Placebo | Delirium |
| Sinha, 1999 | India | NR | 48 | Hospital | Ondansetron, Placebo | Delirium |
| Tsui, 1999 | China | NR | 48 | Hospital | Ondansetron, Placebo, | Mortality |

| | | | | | | |
|---|---|---|---|---|---|---|
| | | | | | Tropisetron | |
| Diemunsch, 1998 | France | NR | 24 | NR | Dolsetron, Placebo | Arrhythmia |
| Goodarzi, 1998 | USA | NR | 48 | NR | Droperidol, Ondansetron, Placebo | Mortality |
| Hamid, 1998 | Canada | NR | 24 | NR | Dimenhydrinate, Ondansetron, Placebo | Arrhythmia |
| Morris, 1998 | Australia, Canada, Denmark, France, Germany, Iceland, Israel, Netherlands, Norway, South Africa, Sweden, UK | NR | 24 | Multi-centre | Metoclopramide, Ondansetron, Placebo | Mortality |
| Scholz, 1998 | Germany | NR | 24 | NR | Ondansetron, Placebo, Tropisetron | Arrhythmia |
| Tramer, 1998 | Switzerland | NR | 48 | Hospital | Ondansetron, Placebo | Arrhythmia |
| Diemunsch, 1997b | France | NR | 24 | NR | Dolsetron, Placebo | Arrhythmia |
| Diemunsch, 1997c | Europe | NR | 24 | NR | Dolsetron, Placebo | Arrhythmia, Mortality |
| Graczyk, 1997 | USA | NR | 24 | NR | Dolsetron, Placebo | QT Interval |
| Klockgether-Radke, 1997 | Germany | NR | 24 | NR | Droperidol, Ondansetron, Placebo | Arrhythmia |
| Koivuranta, 1997 | Finland | NR | 24 | Hospital | Droperidol, Ondansetron, Placebo | Delirium |
| Korttila, 1997 | finland | NR | 24 | Multi-centre | Dolsetron, Ondansetron, Placebo | Arrhythmia |
| Kovac, 1997 | USA | NR | 24 | NR | Dolsetron, Placebo | QT Interval |
| Mikawa, 1997 | Japan | NR | 24 | NR | Granisetron, Placebo | Arrhythmia |
| Monagle, 1997 | Australia | NR | 24 | NR | Metoclopramide, Ondansetron | Arrhythmia |
| Morton, 1997 | Denmark, Sweden, UK, France, Belgium, Germany, Norway. | NR | 24 | Multi-centre | Ondansetron, Placebo | Arrhythmia |
| Patel, 1997 | USA | NR | 24 | Multi-centre | Ondansetron, Placebo | Delirium |
| Purhonen, 1997 | Finland | NR | 48 | Hospital | Droperidol, Placebo, Tropisetron | Arrhythmia |
| Rung, 1997 | USA | NR | 24 | Multi-centre | Ondansetron, Placebo | Mortality |
| Scuderi, 1997 | USA | NR | 24 | NR | Droperidol, Metoclopramide, Ondansetron, Placebo | Arrhythmia |
| Taylor, 1997 | UK | NR | 144 | Multi-centre | Granisetron, Placebo | Mortality, Arrhythmia |
| Ulusoy, 1997 | Turkey | nr | 24 | Hospital | Ondansetron, Placebo | Delirium |
| Warriner, 1997 | Canada | NR | 24 | Multi-centre | Dolsetron, Placebo | Mortality, Arrhythmia |
| Ali-melkkila, 1996 | Finland | NR | 24 | Hospital | Metoclopramide, Placebo, Tropisetron | Mortality |
| Capouet, 1996 | Belgium | NR | 24 | Multi-centre | Placebo, Tropisetron | QT Interval, Arrhythmia |
| Kovac, 1996 (CR: Pearman, 1994c) | USA | NR | 24 | NR | Ondansetron, Placebo | Delirium |
| Naguib, 1996 | Saudi Arabia | NR | 24 | NR | Granisetron, Metoclopramide, Ondansetron, Placebo, Tropisetron | Mortality |
| Rose, 1996a | USA | NR | 24 | NR | Ondansetron, Placebo | Mortality |
| Desilva, 1995 | USA | NR | 4 | NR | Droperidol, Metoclopramide, Ondansetron, Perphenazine, Placebo | Delirium |
| Litman, 1995 | USA | NR | 24 | Hospital | Droperidol, Ondansetron | Delirium |
| Paech, 1995 | Australia | NR | 24 | Hospital | Droperidol, Ondansetron, Placebo | Delirium |
| Paxton, 1995a | Ireland | NR | 24 | NR | Droperidol, Ondansetron, Placebo | Arrhythmia |

| | | | | | | |
|---|---|---|---|---|---|---|
| Kaufmann, 1994 | Switzerland, USA | NR | 35 | NR | Droperidol, Metoclopramide, Placebo, Tropisetron | Delirium |
| Ummenhofer, 1994 | Switzerland | NR | 24 | Hospital | Ondansetron, Placebo | Delirium |
| Dupeyron, 1993 | Belgium, Denmark, France | NR | 24 | NR | Ondansetron, Placebo | Arrhythmia |
| Helmers, 1993 | Netherlands, Ireland, Sweden, UK, South Africa, France, | NR | 24 | NR | Ondansetron, Placebo | Mortality, Arrhythmia |
| Raphael, 1993 | UK | NR | 24 | NR | Metoclopramide, Ondansetron | Delirium |
| Du Pen, 1992 (CR: Scuderi, 1993) | USA | NR | 24 | Multi-centre | Ondansetron, Placebo | Delirium |
| Kovac, 1992 (CRs: McKenzie, 1993a; Pearman, 1994a) | USA | NR | 24 | Multi-centre | Ondansetron, Placebo | Delirium |
| ***Non-randomized clinical trials (n=2)*** | | | | | | |
| Charbit, 2005 | France | NR | 3 | Hospital | Droperidol, Ondansetron | QT prolongation, Arrhythmia |
| Lerman, 1996 | Canada | NR | 24 | Hospital | Dolasetron | QT prolongation |
| ***Controlled before-after studies (n=1)*** | | | | | | |
| Wagner, 2007 | USA | NR | 24 | Medical center | Ondansetron, Placebo | Delirium |
| ***Cohort studies (n=0)*** | | | | | | |

**Note:** [†]Unpublished data (White, 2005).
**Abbreviations:** CR, companion report; NR, not reported; UK, United Kingdom; USA United States of America.

## Appendix 3. Frequencies for specific drugs and drug combinations

| ***Intervention/ Comparator:*** | **% of arms (n=367)** | **% of studies (n=115)** |
|---|---|---|
| Ondansetron | 26.16 | 68.7 |
| Placebo | 23.71 | 74.78 |
| Dolasetron | 10.62 | 13.04 |
| Granisetron | 5.99 | 12.17 |
| Droperidol | 5.72 | 20 |
| Tropisetron | 5.45 | 13.04 |
| Metoclopramide | 3.81 | 12.17 |
| Palonosetron | 3.27 | 3.48 |
| Ondansetron + Dexamethasone | 1.91 | 6.09 |
| Dexamethasone | 1.63 | 5.22 |
| Ramosetron | 1.36 | 2.61 |
| Aprepitant | 1.09 | 1.74 |
| Granisetron + Dexamethasone | 1.09 | 3.48 |
| Haloperidol | 1.09 | 3.48 |
| Rolapitant | 1.09 | 0.87 |
| Ondansetron + Casopitant | 0.82 | 0.87 |
| Ondansetron + Droperidol | 0.82 | 2.61 |
| Dimenhydrinate | 0.54 | 1.74 |
| Haloperidol + Dexamethasone | 0.54 | 1.74 |
| Casopitant | 0.27 | 0.87 |
| Cyclizine | 0.27 | 0.87 |
| Dolasetron + Dexamethasone | 0.27 | 0.87 |
| Dolasetron + Droperidol | 0.27 | 0.87 |
| Droperidol + Dexamethasone | 0.27 | 0.87 |
| Granisetron + Droperidol | 0.27 | 0.87 |
| Metoclopramide + Dexamethasone | 0.27 | 0.87 |
| Ondansetron + Cyclizine | 0.27 | 0.87 |
| Ondansetron + Haloperidol | 0.27 | 0.87 |
| Perphenazine | 0.27 | 0.87 |
| Promethazine | 0.27 | 0.87 |
| Ramosetron + Dexamethasone | 0.27 | 0.87 |

## Appendix 4. Patient characteristics

| Author, year | Sample size | Age Category | % female | ASA status | Surgery type | H/o motion sickness | H/o PONV | Comorbidities |
|---|---|---|---|---|---|---|---|---|
| ***Randomized clinical trials (n=112)*** | | | | | | | | |
| Choi, 2012 | 120 | Adults | 100 | I or II | Orthopaedic | YES | YES | NR |
| Wang, 2012 | 130 | Adults | 100 | I or II | Miscellaneous | YES | YES | NR |
| Ekinci, 2011 | 100 | Adults | 100 | I or II or III | Obstetrics & Gynaecological | NR | NR | NR |
| El-deeb, 2011b | 160 | Adults | 100 | I or II | Obstetrics & Gynaecological | NR | NR | NR |
| Gan, 2011 | 619 | Adults | 100 | I or II or III | Miscellaneous | YES | YES | NR |
| Gupta, 2011 | 136 | Adults | 62 | I or II | Miscellaneous | NR | NR | NR |
| Park, 2011 | 90 | Adults | 100 | I or II | Obstetrics & Gynaecological | YES | YES | NR |
| Ryu, 2011 | 124 | Adults | 54 | I or II | Gastrointestinal | NR | NR | Cardiovascular, liver disease |
| Sahoo, 2011 | 52 | Adults | 100 | I | Obstetrics & Gynaecological | NR | NR | NR |
| Bilgin, 2010 | 160 | Adults | 100 | I or II | Obstetrics & Gynaecological | NR | NR | NR |
| Choi, 2010 | 279 | Adults & Elderly | 39 | NR | Cardiovascular | YES | YES | NR |
| Jee, 2010 | 100 | Adults | 100 | I or II | Obstetrics & Gynaecological | YES | YES | NR |
| Jokela, 2010 | 80 | Adults | 100 | I or II or III | Obstetrics & Gynaecological | YES | YES | NR |
| Mehta, 2010 | 80 | Children | 50 | I or II | Miscellaneous | NR | NR | NR |
| Shakya, 2010 | 80 | Adults | 63 | I or II | Miscellaneous | NR | NR | NR |
| Singla, 2010 | 702 | Adults | 100 | NR | Miscellaneous | YES | YES | NR |
| Feng, 2009 | 192 | Adults | 74 | I or II | Gastrointestinal | YES | YES | NR |
| Jain, 2009 | 87 | Adults | 29.9 | I or II | Neurological | NR | NR | NR |
| Riad, 2009 | 100 | Children | 48 | I or II | Eye | NR | NR | NR |
| Rosow, 2009 | 244 | Adults & Elderly | 66 | I or II or III | Miscellaneous | NR | NR | NR |
| Candiotti, 2008 | 546 | Adults & Elderly | 95 | I or II or III | Miscellaneous | YES | YES | NR |
| Choi, 2008 | 94 | Adults | 100 | NR | Orthopaedic | YES | YES | NR |
| Contreras-Dominguez, 2008 | 150 | Adults | 49 | I or II | Gastrointestinal | NR | YES | NR |
| Kovac, 2008 | 544 | Adults | 100 | I or II or III | Miscellaneous | YES | YES | NR |
| Owczuk, 2008 | 71 | Adults | 11 | I or II | Miscellaneous | NR | NR | NR |
| Piper, 2008 | 83 | Adults | 56 | I or II | General dentistry, oral and maxillofacial surgery and orthodontics | YES | YES | NR |
| Said-Ahmed, 2008 | 50 | Adults | 100 | I or II | Obstetrics & Gynaecological | NR | NR | NR |
| Bestas, 2007 | 90 | Adults | 77 | I or II | Gastrointestinal | NR | NR | NR |
| Diemunsch, 2007 | 892 | Adults & Elderly | 91 | I or II or III | Miscellaneous | YES | YES | NR |
| Gan, 2007 | 766 | Adults & Elderly | 94 | I or II or III | Miscellaneous | YES | YES | NR |
| Han, 2007 | 80 | Adults | 100 | I | Obstetrics & Gynaecological | NR | NR | NR |
| Lee, 2007 | 86 | Adults | 100 | I or II | Obstetrics & Gynaecological | YES | YES | NR |
| Oksuz, 2007 | 75 | Adults & Elderly | 65 | I or II | Gastrointestinal | NR | YES | NR |
| Rusch, 2007 | 228 | Adults & Elderly | 49 | I or II or III | Miscellaneous | NR | YES | NR |
| Sagir, 2007 | 80 | Adults | 0.7 | I or II | Urological | NR | NR | NR |
| Sandhu, 2007 | 80 | Adults | 71 | I or II | Gastrointestinal | NR | NR | NR |
| Siddik-Sayyid, 2007 | 129 | Adults | 100 | I or II | Obstetrics & | NR | NR | Obesity |

| | | | | | | | | |
|---|---|---|---|---|---|---|---|---|
| | | | | | Gynaecological | | | |
| Bridge, 2006 | 194 | Adults | 100 | NR | Miscellaneous | NR | NR | NR |
| Chan, 2006 | 394 | Adults | 100 | I or II | Obstetrics & Gynaecological | YES | YES | NR |
| Kelsaka, 2006 | 50 | Adults | 25 | I or II | Orthopaedic | NR | NR | NR |
| Sarvela, 2006 | 87 | Adults | 100 | NR | Obstetrics & Gynaecological | NR | NR | NR |
| Tosun, 2006 | 125 | Children | 49 | I or II | Eye | NR | NR | NR |
| D'Angelo, 2005 | 121 | Adults | 100 | I or II or III | Obstetrics & Gynaecological | YES | YES | NR |
| Gan, 2005 | 176 | Adults | 100 | I or II or III | Obstetrics & Gynaecological | YES | YES | NR |
| Khalil, 2005 | 670 | Children | 24 | I or II or III | Miscellaneous | YES | YES | NR |
| Kocamanoglu, 2005 | 150 | Adults | 100 | I or II | Obstetrics & Gynaecological | YES | YES | NR |
| Kontrimaviciute, 2005 | 34 | Adults | 100 | I or II | Obstetrics & Gynaecological | NR | YES | NR |
| Pirat, 2005 | 150 | Adults | 0 | I | Miscellaneous | NR | NR | NR |
| Treschan, 2005 | 142 | All groups | 56 | I or II | Eye | YES | YES | NR |
| White, 2005† | 372 | NR | 100 | NR | Obstetrics & Gynaecological | NR | NR | NR |
| Apfel, 2004 (CR: Jokela, 2009) | 260 | Adults | 81 | NR | Miscellaneous | YES | YES | NR |
| Binstock, 2004 | 101 | Children | 42 | I or II | Miscellaneous | NR | NR | NR |
| Eberhart, 2004 | 304 | Adults & Elderly | 52 | I or II or III | Eye | YES | YES | NR |
| Hanaoka, 2004 | 315 | Adults | 95 | I or II or III | Miscellaneous | YES | NR | NR |
| Samakarndi, 2004 | 80 | Children | 49 | I or II | Eye | NR | NR | NR |
| Charuluxananan, 2003 | 180 | Adults | 100 | I or II | Obstetrics & Gynaecological | NR | NR | NR |
| Loewen, 2003 | 71 | Adults | 100 | NR | Breast | YES | YES | NR |
| O'Brien, 2003 | 150 | Children | NR | I or II | Miscellaneous | YES | YES | NR |
| Argiriadou, 2002 | 87 | Adults | 76 | I or II | Gastrointestinal | NR | NR | NR |
| Gurkan, 2002 | 150 | Adults | 53 | I or II | Miscellaneous | NR | NR | NR |
| Dabbous, 2001 | 173 | Adults | 77 | I or II | Gastrointestinal | YES | YES | Diabetes |
| Kathirvel, 2001 | 152 | Adults & Children | 38 | I or II | Neurological | NR | NR | NR |
| Subramaniam, 2001 | 135 | Children | 54 | I or II | Eye | NR | YES | NR |
| Ahmed, 2000 | 139 | Adults | 100 | NR | Obstetrics & Gynaecological | YES | YES | NR |
| Charuluxananan, 2000 | 80 | Adults | 100 | I or II | Obstetrics & Gynaecological | NR | NR | NR |
| Jensen, 2000 | 71 | Children | 41 | I or II | General dentistry, oral and maxillofacial surgery and orthodontics | YES | NR | NR |
| Kreisler, 2000 | 31 | Adults | 81 | NR | Miscellaneous | NR | YES | NR |
| Philip, 2000 | 1030 | Adults | 70 | I or II or III | Miscellaneous | YES | YES | NR |
| Zarate, 2000 | 200 | Adults | 44 | I or II | Miscellaneous | YES | YES | NR |
| Koivuranta, 1999 | 428 | Adults | 82 | I or II or III | Miscellaneous | YES | YES | NR |
| McCall, 1999 | 73 | Children | 53 | I or II | Eye | NR | NR | NR |
| Sinha, 1999 | 40 | Adults | 47 | I or II or III | Neurological | NR | YES | NR |
| Tsui, 1999 | 121 | Adults | 100 | I or II or III | Obstetrics & Gynaecological | NR | NR | NR |
| Diemunsch, 1998 | 793 | Adults | 100 | I or II or III | Obstetrics & Gynaecological | YES | YES | NR |
| Goodarzi, 1998 | 81 | Children | 43 | I or II | Orthopaedic | NR | NR | NR |
| Hamid, 1998 | 71 | Children | 55 | I or II | General dentistry, oral and maxillofacial surgery and orthodontics | NR | YES | NR |
| Morris, 1998 | 1044 | Adults | 100 | I or II or III | Obstetrics & Gynaecological | NR | YES | NR |
| Scholz, 1998 | 842 | Adults & | 74 | I or II | Miscellaneous | YES | YES | NR |

| | | | | | | | | |
|---|---|---|---|---|---|---|---|---|
| | | Elderly | | or III | | | | |
| Tramer, 1998 | 157 | Children | 49 | NR | Eye | YES | YES | |
| Diemunsch, 1997b | 281 | Adults | 100 | I or II | Obstetrics & Gynaecological | NR | YES | NR |
| Diemunsch, 1997c | 337 | Adults | 95 | I or II | Obstetrics & Gynaecological | NR | YES | NR |
| Graczyk, 1997 | 635 | Adults | 100 | I or II | Obstetrics & Gynaecological | NR | YES | NR |
| Klockgether-Radke, 1997 | 120 | Children | 48 | I or II | Eye | NR | NR | NR |
| Koivuranta, 1997 | 439 | Adults & Elderly | 100 | I or II or III | Miscellaneous | YES | YES | Migraines |
| Korttila, 1997 | 517 | Adults | 94 | I or II or III | Miscellaneous | YES | YES | NR |
| Kovac, 1997 | 620 | Adults | 83 | I or II or III | Miscellaneous | YES | YES | NR |
| Mikawa, 1997 | 200 | Adults & Elderly | 100 | I | Obstetrics & Gynaecological | NR | YES | NR |
| Monagle, 1997 | 91 | Adults | 100 | NR | Obstetrics & Gynaecological | NR | YES | NR |
| Morton, 1997 | 427 | Children | 51 | I or II | General dentistry, oral and maxillofacial surgery and orthodontics | NR | YES | NR |
| Patel, 1997 | 429 | Children | 37 | I or II or III | Miscellaneous | YES | YES | NR |
| Purhonen, 1997 | 146 | Adults | 100 | I or II or III | Miscellaneous | YES | YES | NR |
| Rung, 1997 | 121 | Adults & Elderly | 69 | I or II or III | Miscellaneous | YES | YES | NR |
| Scuderi, 1997 | 160 | Children | 52 | I or II | Eye | NR | YES | NR |
| Taylor, 1997 | 523 | Adults & Elderly | 89 | I or II or III | Multiple types included | YES | YES | NR |
| Ulusoy, 1997 | 40 | Adults | 37 | I or II | Miscellaneous | NR | NR | NR |
| Warriner, 1997 | 374 | Adults & Elderly | 100 | I or II | Obstetrics & Gynaecological | YES | YES | NR |
| Ali-melkkila, 1996 | 120 | Adults | 42 | I or II or III | Eye | NR | YES | NR |
| Capouet, 1996 | 385 | Adults | 100 | I or II | Miscellaneous | YES | YES | NR |
| Kovac, 1996 (CR: Pearman, 1994c) | 30 | Adults | 100 | I or II | Obstetrics & Gynaecological | NR | NR | NR |
| Naguib, 1996 | 48 | Adults | 100 | I or II | Obstetrics & Gynaecological | NR | NR | NR |
| Rose, 1996a | 136 | Children | 44 | I or II | General dentistry, oral and maxillofacial surgery and orthodontics | NR | NR | NR |
| Desilva, 1995 | 50 | Adults | 70 | I or II | Gastrointestinal | NR | NR | NR |
| Litman, 1995 | 57 | Children | 40 | I or II | Eye | YES | YES | NR |
| Paech, 1995 | 259 | Adults | 100 | NR | Obstetrics & Gynaecological | NR | YES | NR |
| Paxton, 1995a | 60 | Children | 47 | NR | Ear, nose and larynx | NR | NR | NR |
| Kaufmann, 1994 | 286 | Adults | 54 | I or II | Orthopaedic | NR | NR | NR |
| Ummenhofer, 1994 | 200 | Children | 34 | I or II | Miscellaneous | NR | NR | NR |
| Dupeyron, 1993 | 243 | Adults & Elderly | 100 | I or II or III | Obstetrics & Gynaecological | NR | NR | Cardiovascular, urological |
| Helmers, 1993 | 923 | Adults | 100 | I or II or III | Obstetrics & Gynaecological | NR | NR | NR |
| Raphael, 1993 | 123 | Adults | 100 | NR | Obstetrics & Gynaecological | YES | YES | NR |
| Du Pen, 1992 (CR: Scuderi, 1993) | 500 | All groups | 11 | I or II | Miscellaneous | NR | NR | NR |
| Kovac, 1992 (CRs: McKenzie, 1993a; Pearman, 1994a) | 580 | Adults | 100 | I or II | Obstetrics & Gynaecological | NR | YES | NR |
| ***Non-randomized clinical trials (n=2)*** | | | | | | | | |
| Charbit, 2005 | 85 | Adults | 60 | NR | Miscellaneous | NR | NR | NR |

| | | | | | | | | |
|---|---|---|---|---|---|---|---|---|
| Lerman, 1996 | 28 | Children | 32 | I or II | Otolaryngological | NR | NR | NR |
| ***Controlled before-after studies (n=1)*** | | | | | | | | |
| Wagner, 2007 | 66 | Children | 54 | I or II | Eye | NR | NR | NR |

**Note:** †Unpublished data (White, 2005).

**Abbreviations:** ASA, American Society of Anaesthesiologists physical classification system; NR, not reported or No; CR, companion report; PONV, post-operative nausea and vomiting; H/o, History of.

## Appendix 5. Cochrane EPOC Risk-of-bias appraisal results

| Author, year | 1 | 2 | 3 | 4 | 5 | 6 | 7 | 8 | 9 |
|---|---|---|---|---|---|---|---|---|---|
| ***Randomized clinical trials (n=112)*** | | | | | | | | | |
| Choi, 2012 | Low | Low | Unclear | Low | Unclear | Low | Low | Unclear | Unclear |
| Wang, 2012 | Low | High | Unclear | Low | Low | Low | Unclear | Unclear | Unclear |
| Ekinci, 2011 | Low | Unclear | Unclear | Low | Low | Low | Low | Unclear | Unclear |
| El-deeb, 2011b | Low | Low | Unclear | Low | Unclear | Low | Low | Unclear | Unclear |
| Gan, 2011 | Low | Low | Unclear | Low | High | Low | Unclear | Low | Low |
| Gupta, 2011 | Low | Unclear | Unclear | Unclear | Unclear | Unclear | Low | Unclear | Unclear |
| Park, 2011 | Low | Unclear | Unclear | Low | Low | Low | Low | Unclear | Low |
| Ryu, 2011 | Low | Low | Unclear | Low | Low | Low | Low | Low | Low |
| Sahoo, 2011 | Low | Unclear | Unclear | Low | Unclear | Low | Low | Unclear | Unclear |
| Bilgin, 2010 | Low | Low | Unclear | Low | Low | Low | Low | Unclear | Unclear |
| Choi, 2010 | Low | Low | Low | Low | High | Unclear | Low | Unclear | Unclear |
| Jee, 2010 | Low | Unclear | Unclear | Low | Unclear | Unclear | Unclear | Unclear | Unclear |
| Jokela, 2010 | Low | Unclear | Unclear | Low | High | Low | Low | Unclear | Unclear |
| Mehta, 2010 | Low | Low | Low | Low | High | Low | Low | Unclear | Unclear |
| Shakya, 2010 | Unclear | Unclear | Unclear | Low | Unclear | Unclear | Unclear | Unclear | Low |
| Singla, 2010 | Unclear | Unclear | Unclear | Low | Low | Low | Unclear | Low | Unclear |
| Feng, 2009 | Low | Low | Low | Low | Low | Low | Low | Unclear | Low |
| Jain, 2009 | Low | Unclear | Unclear | Low | High | Unclear | Unclear | Unclear | Unclear |
| Riad, 2009 | Unclear | Low | Unclear | Low | Low | Low | Low | Unclear | Low |
| Rosow, 2009 | Unclear | High | Unclear | Low | Low | Low | Unclear | Unclear | High |
| Candiotti, 2008 | Low | Low | Low | Low | Low | Unclear | Low | Unclear | Unclear |
| Choi, 2008 | Low | Low | Unclear | Low | Unclear | Low | Low | Unclear | Unclear |
| Contreras-Dominguez, 2008 | Unclear | Unclear | Unclear | Low | Unclear | Low | Low | Unclear | Unclear |
| Kovac, 2008 | Low | Unclear | Unclear | Low | Unclear | Unclear | Low | Unclear | Unclear |
| Owczuk, 2008 | Low | Unclear | Unclear | Low | Low | Unclear | Low | Unclear | Unclear |
| Piper, 2008 | Unclear | Low | Unclear | Low | Unclear | Low | Low | Low | Low |
| Said-Ahmed, 2008 | Unclear | Low | Unclear | Low | Unclear | Low | Low | Unclear | Unclear |
| Bestas, 2007 | Unclear | Unclear | Unclear | Low | Low | Low | Low | Unclear | Unclear |
| Diemunsch, 2007 | Low | Low | Unclear | Low | Unclear | Low | Unclear | Low | High |
| Gan, 2007 | Low | Low | Unclear | Low | Low | Low | Low | Unclear | Unclear |
| Han, 2007 | Unclear | Unclear | Unclear | Low | Low | Low | Unclear | Unclear | Unclear |
| Lee, 2007 | Low | Low | Low | Low | Low | Low | Low | Unclear | Low |
| Oksuz, 2007 | Unclear | Unclear | Unclear | Low | Low | Low | Low | Unclear | Low |
| Rusch, 2007 | Low | Unclear | Low | Low | Unclear | Low | Unclear | Unclear | Low |
| Sagir, 2007 | Low | Low | Unclear | Low | Unclear | Low | Low | Unclear | Unclear |
| Sandhu, 2007 | Unclear | Low | Unclear | Low | Low | Low | Low | Unclear | Unclear |
| Siddik-Sayyid, 2007 | Low | Low | Unclear | Low | Low | Low | Low | Unclear | Low |
| Bridge, 2006 | Unclear | Low | Unclear | Low | Unclear | Unclear | Low | Unclear | Unclear |
| Chan, 2006 | Low | Low | Low | Low | Low | Low | Low | Unclear | Low |
| Kelsaka, 2006 | Unclear | Unclear | Unclear | Low | Unclear | Low | Low | Unclear | Unclear |
| Sarvela, 2006 | Unclear | Low | Low | Low | High | Low | Low | Unclear | Unclear |
| Tosun, 2006 | Unclear | Unclear | Unclear | Low | Unclear | Unclear | Unclear | Unclear | Unclear |
| D'Angelo, 2005 | Low | Low | Unclear | High | Low | Low | Low | Unclear | Unclear |
| Gan, 2005 | Low | Low | Unclear | Low | Low | Low | Low | Unclear | Unclear |
| Khalil, 2005 | Unclear | High | Low | Low | High | Unclear | Unclear | Unclear | High |
| Kocamanoglu, 2005 | Unclear | Unclear | Low | Low | Unclear | Low | Low | Unclear | Unclear |
| Kontrimaviciute, 2005 | Unclear | Unclear | Unclear | Low | Unclear | Unclear | Unclear | Unclear | Unclear |
| Pirat, 2005 | Unclear | Unclear | Unclear | Low | Unclear | Low | Low | Unclear | Unclear |
| Treschan, 2005 | Low | Low | Unclear | Low | Unclear | Low | Low | Unclear | Unclear |
| White, 2005† | Unclear | Unclear | Unclear | Low | Unclear | Unclear | Unclear | Unclear | Unclear |
| Apfel, 2004 (CR: Jokela, 2009) | Low | Low | Unclear | Unclear | Low | Low | Low | Unclear | Low |
| Binstock, 2004 | Unclear | Unclear | Unclear | High | Low | Unclear | Low | Unclear | Unclear |
| Eberhart, 2004 | Unclear | Low | Unclear | Low | Low | Low | Low | Unclear | Unclear |
| Hanaoka, 2004 | Unclear | Low | Unclear | Low | High | Unclear | Unclear | Unclear | Unclear |
| Samakarndi, 2004 | Unclear | Unclear | Low | Unclear | Low | Low | Low | Unclear | Unclear |
| Charuluxananan, 2003 | Low | Low | Unclear | Low | Low | Low | Low | Unclear | Unclear |

| | | | | | | | | | |
|---|---|---|---|---|---|---|---|---|---|
| Loewen, 2003 | Low | Low | Unclear | Low | Low | Low | Low | Unclear | Unclear |
| O'Brien, 2003 | Unclear | Unclear | Unclear | Unclear | Low | Unclear | Low | Unclear | Unclear |
| Argiriadou, 2002 | Low | Low | Unclear | Low | Unclear | Low | Low | Unclear | Unclear |
| Gurkan, 2002 | Low | Unclear | Unclear | Low | Unclear | Low | Low | Unclear | Unclear |
| Dabbous, 2001 | Low | Low | Unclear | Low | High | Low | Low | Unclear | Unclear |
| Kathirvel, 2001 | Low | Unclear | Unclear | Low | Unclear | Low | Low | Unclear | Unclear |
| Subramaniam, 2001 | Low | Low | Unclear | Low | Low | Low | Low | Unclear | Unclear |
| Ahmed, 2000 | Low | Unclear | Unclear | Low | Low | Low | Low | Unclear | Unclear |
| Charuluxananan, 2000 | Low | Low | Unclear | Low | Unclear | Unclear | Low | Unclear | Unclear |
| Jensen, 2000 | Low | Unclear | Unclear | Unclear | Low | Low | Low | Unclear | Low |
| Kreisler, 2000 | Unclear | Unclear | Unclear | Low | High | Low | Low | Unclear | High |
| Philip, 2000 | Unclear | Unclear | Unclear | Low | Low | Unclear | Low | Unclear | Low |
| Zarate, 2000 | Low | Unclear | Unclear | Low | Low | Low | Unclear | Unclear | Unclear |
| Koivuranta, 1999 | Unclear | Low | Unclear | Low | Low | Low | Low | Unclear | Unclear |
| McCall, 1999 | Unclear | Unclear | Unclear | Low | Unclear | Low | Low | Unclear | Unclear |
| Sinha, 1999 | Low | Unclear | Unclear | Low | High | Low | Low | Unclear | Unclear |
| Tsui, 1999 | Unclear | Unclear | Unclear | Low | Unclear | Low | Unclear | Unclear | Low |
| Diemunsch, 1998 | Unclear | Unclear | Unclear | Low | Low | Unclear | Low | Unclear | Unclear |
| Goodarzi, 1998 | Unclear | Unclear | Unclear | Low | Low | Low | Low | Unclear | Unclear |
| Hamid, 1998 | Low | Low | Unclear | Low | Low | Low | Low | Unclear | Unclear |
| Morris, 1998 | Unclear | Unclear | Unclear | Low | Unclear | Unclear | Unclear | Unclear | Unclear |
| Scholz, 1998 | Unclear | Low | Unclear | Low | Low | Unclear | Unclear | Unclear | Unclear |
| Tramer, 1998 | Low | Unclear | Unclear | Low | Unclear | Low | Low | Unclear | Unclear |
| Diemunsch, 1997b | Unclear | Low | Low | Low | Unclear | Unclear | Low | Unclear | Unclear |
| Diemunsch, 1997c | Unclear | Unclear | Low | Low | Unclear | Unclear | Low | Unclear | Unclear |
| Graczyk, 1997 | Unclear | Low | Unclear | Low | Low | Low | Low | Unclear | Unclear |
| Klockgether-Radke, 1997 | Unclear | Unclear | Unclear | Low | Unclear | Unclear | Low | Unclear | Unclear |
| Koivuranta, 1997 | Low | Unclear | Unclear | High | Low | Low | Low | Unclear | Unclear |
| Korttila, 1997 | Unclear | Unclear | Unclear | Low | Low | Unclear | Unclear | Unclear | Unclear |
| Kovac, 1997 | Low | Low | Low | High | Low | Unclear | Low | Unclear | Unclear |
| Mikawa, 1997 | Unclear | Unclear | Low | Low | Unclear | Low | Low | Unclear | Unclear |
| Monagle, 1997 | Unclear | Low | Low | Low | Low | Low | Unclear | Unclear | Unclear |
| Morton, 1997 | Unclear | Unclear | Unclear | Low | Low | Unclear | Low | Unclear | Unclear |
| Patel, 1997 | Unclear | Unclear | Unclear | Low | Low | Low | Low | Unclear | Unclear |
| Purhonen, 1997 | Unclear | Low | Low | Low | Low | Low | Unclear | Unclear | Unclear |
| Rung, 1997 | Low | Low | Low | Low | Unclear | Unclear | Low | Unclear | Unclear |
| Scuderi, 1997 | Low | Unclear | Unclear | Low | Unclear | Unclear | Low | Unclear | Unclear |
| Taylor, 1997 | Low | Unclear | Low | Low | High | Unclear | Unclear | Unclear | Unclear |
| Ulusoy, 1997 | Unclear | Unclear | Unclear | Unclear | Unclear | Unclear | Low | Unclear | Unclear |
| Warriner, 1997 | Unclear | Unclear | Unclear | Low | Low | Unclear | Low | Unclear | Unclear |
| Ali-melkkila, 1996 | Unclear | Unclear | Unclear | Low | Low | Unclear | Low | Unclear | Unclear |
| Capouet, 1996 | Unclear | Low | Unclear | Low | Unclear | Unclear | Low | Unclear | Unclear |
| Kovac, 1996 (CR: Pearman, 1994c) | Unclear | Unclear | Unclear | Low | Unclear | Unclear | Low | Unclear | Unclear |
| Naguib, 1996 | Unclear | Low | Unclear | Low | Unclear | Low | Low | Unclear | Unclear |
| Rose, 1996a | Unclear | Low | Unclear | Low | Low | Low | Low | Unclear | Unclear |
| Desilva, 1995 | Unclear | Low | Low | Low | High | Low | Low | Unclear | Unclear |
| Litman, 1995 | Low | Unclear | Unclear | Low | Unclear | Unclear | Unclear | Unclear | Unclear |
| Paech, 1995 | Low | Unclear | Unclear | Low | Low | Unclear | Low | Unclear | Unclear |
| Paxton, 1995a | Unclear | Unclear | Unclear | Low | Unclear | Unclear | Low | Unclear | Low |
| Kaufmann, 1994 | Low | Low | Unclear | High | Low | Low | Low | Unclear | Unclear |
| Ummenhofer, 1994 | Low | Low | Unclear | Low | Unclear | Low | Low | Unclear | Unclear |
| Dupeyron, 1993 | Unclear | Unclear | Unclear | Low | Unclear | Unclear | Low | Unclear | Unclear |
| Helmers, 1993 | Unclear | Unclear | Unclear | Low | Low | Unclear | Unclear | Unclear | Unclear |
| Raphael, 1993 | Unclear | Unclear | Low | Low | Low | Unclear | Unclear | Unclear | Unclear |
| Du Pen, 1992 (CR: Scuderi, 1993) | Unclear | Unclear | Low | High | Unclear | Unclear | Low | Unclear | Unclear |
| Kovac, 1992 (CRs: McKenzie, 1993a; Pearman, 1994a) | Unclear | Unclear | Low | Low | Low | Unclear | Low | Unclear | Unclear |
| ***Non-randomized clinical trials (n=2)*** | | | | | | | | | |
| Charbit, 2005 | High | High | Low | Low | Unclear | Low | Low | Unclear | Unclear |
| Lerman, 1996 | High | High | Unclear | Low | Unclear | High | Low | Unclear | Unclear |

| | | | | | | | | | |
|---|---|---|---|---|---|---|---|---|---|
| ***Controlled before-after studies (n=1)*** | | | | | | | | | |
| Wagner, 2007 | High | High | Unclear | High | High | High | Unclear | Unclear | High |

**Note:** †Unpublished data.
**Abbreviations:** CR, companion report; EPOC, Effective Practice and Organization of Care; High, high risk; Low, low risk; Unclear, unclear risk .
**Items:**

1. Random sequence generation
2. Allocation concealment
3. Similar baseline outcome measures
4. Similar baseline characteristics
5. Incomplete outcome data
6. Blinding of outcome assessor
7. Contamination
8. Selective outcome reporting
9. Other bias

**Appendix 6. Aggregate EPOC risk-of-bias appraisal results**

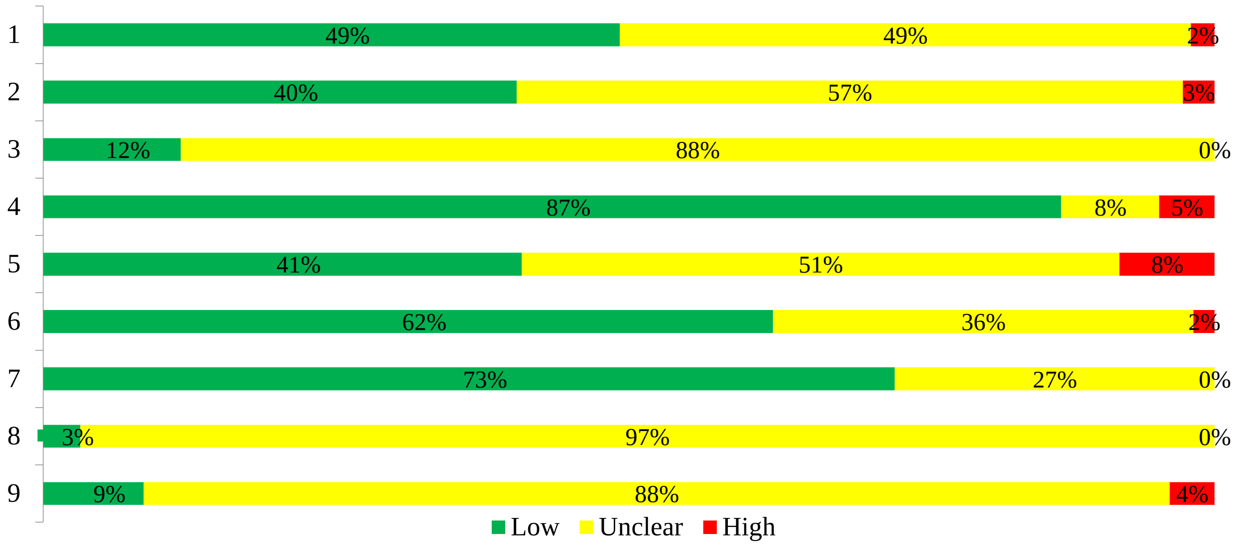


**Abbreviations:** EPOC, Effective Practice and Organization of Care; High, High risk of bias; Low, Low risk of bias; Unclear, Unclear risk of bias.

**Items:**

1. Random sequence generation
2. Allocation concealment
3. Similar baseline outcome measures
4. Similar baseline characteristics
5. Incomplete outcome data
6. Blinding
7. Contamination
8. Selective outcome reporting
9. Other bias

## Appendix 7. McHarm appraisal results

| Author, year | 1 | 2 | 3 | 4 | 5 | 6 | 7 | 8 | 9 | 10 | 11 | 12 | 13 | 14 | 15 |
|---|---|---|---|---|---|---|---|---|---|---|---|---|---|---|---|
| ***Randomized clinical trials*** | | | | | | | | | | | | | | | |
| Wang, 2012 | P | U | U | U | P | U | U | U | P | C | P | C | U | C | U |
| Ekinci, 2011 | P | U | U | U | P | U | U | U | U | P | C | U | U | C | C |
| El-deeb, 2011b | P | U | U | U | P | U | U | U | U | U | U | U | U | C | P |
| Sahoo, 2011 | U | U | U | U | C | U | U | U | C | P | C | U | U | C | P |
| Choi, 2010 | P | U | U | U | P | U | P | U | U | P | P | P | U | P | U |
| Jokela, 2010 | P | U | U | U | C | U | C | C | C | C | C | C | C | C | C |
| Mehta, 2010 | P | U | U | U | C | U | C | C | C | C | U | C | U | U | U |
| Shakya, 2010 | P | U | U | U | U | U | U | U | U | P | P | U | U | C | U |
| Feng, 2009 | P | U | U | U | C | U | U | U | C | P | C | C | U | C | P |
| Jain, 2009 | P | U | U | U | C | U | P | P | P | P | C | C | U | C | U |
| Riad, 2009 | U | U | U | U | C | U | P | U | C | P | U | U | U | C | P |
| Candiotti, 2008 | U | U | U | U | U | U | U | U | U | U | C | C | P | C | U |
| Contreras-Dominguez, 2008 | P | U | U | U | C | U | C | C | C | P | C | U | U | C | U |
| Kovac, 2008 | U | U | U | U | U | U | U | U | U | U | C | U | C | C | C |
| Owczuk, 2008 | P | U | U | U | C | U | U | U | C | P | U | C | U | U | P |
| Piper, 2008 | U | U | U | U | U | U | U | U | U | U | C | U | U | C | U |
| Said-Ahmed, 2008 | P | U | U | U | C | U | C | P | C | P | C | U | U | U | P |
| Aouad, 2007 | P | U | U | U | P | U | C | P | C | C | C | C | U | C | C |
| Balki, 2007 | P | U | U | U | C | U | U | U | P | P | C | C | U | C | C |
| Diemunsch, 2007 | P | U | U | U | C | U | P | U | C | P | C | C | C | C | C |
| Gan, 2007 | P | U | U | C | C | U | P | U | C | C | C | C | C | C | P |
| Han, 2007 | P | U | U | U | P | U | C | C | P | C | U | C | U | C | U |
| Lee, 2007 | P | U | U | U | P | U | C | C | C | P | P | C | U | C | U |
| Rusch, 2007 | U | U | U | U | C | U | P | P | C | P | C | C | C | C | P |
| Sagir, 2007 | C | U | U | U | P | U | U | U | U | P | C | U | U | C | C |
| Siddik-Sayyid, 2007 | P | U | U | U | P | U | C | C | C | P | P | U | U | P | U |
| Chan, 2006 | P | U | U | U | C | U | P | U | C | P | C | C | U | C | P |
| Kelsaka, 2006 | U | U | U | U | U | U | U | U | C | U | U | U | U | U | U |
| Sarvela, 2006 | U | U | U | U | C | P | U | U | C | P | U | C | U | U | P |
| Tosun, 2006 | P | U | U | U | C | NA | C | C | C | U | C | U | U | C | C |
| Gan, 2005 | P | U | U | C | P | U | C | P | U | U | C | C | C | C | P |
| Khalil, 2005 | P | U | U | U | C | NA | P | U | C | U | C | P | P | C | C |
| Kontrimaviciute, 2005 | U | U | U | U | P | U | U | U | P | P | C | U | U | C | U |
| Pirat, 2005 | C | U | U | U | P | U | C | C | C | P | C | U | U | C | U |
| Treschan, 2005 | U | U | U | U | C | U | U | U | C | P | C | U | U | U | P |
| Binstock, 2004 | P | U | U | U | C | U | U | U | C | U | C | P | U | P | C |
| Eberhart, 2004 | P | U | U | U | U | U | U | U | U | U | C | C | U | C | U |
| Hanaoka, 2004 | P | C | U | U | P | U | C | U | P | P | U | C | C | C | U |
| Samakarndi, 2004 | U | U | U | U | C | U | U | U | C | P | U | U | U | U | P |
| Charuluxananan, 2003 | P | U | U | U | C | U | P | U | C | P | C | C | C | C | C |
| Loewen, 2003 | P | U | U | U | C | U | C | C | U | C | C | C | U | C | U |
| O'Brien, 2003 | P | U | U | U | P | U | C | C | C | U | P | C | C | C | U |
| Gurkan, 2002 | P | U | U | U | P | U | U | U | C | P | U | U | U | C | P |
| Dabbous, 2001 | P | U | U | U | U | U | U | U | U | U | C | U | U | C | U |
| Kathirvel, 2001 | P | U | U | U | P | U | C | C | P | U | C | C | C | C | U |
| Subramaniam, 2001 | U | U | U | U | C | U | U | U | C | P | C | U | U | C | P |
| Ahmed, 2000 | U | U | U | U | C | U | C | U | C | U | C | C | C | U | P |
| Charuluxananan, 2000 | U | U | U | U | U | U | P | U | P | U | U | U | U | U | P |
| Jensen, 2000 | U | U | U | U | C | U | U | U | C | P | C | C | U | C | P |
| Kreisler, 2000 | P | U | U | U | P | U | P | U | U | U | C | U | U | C | U |
| Philip, 2000 | P | U | U | U | P | U | U | U | C | P | C | U | U | C | C |
| Zarate, 2000 | P | U | U | U | C | U | C | C | C | U | U | U | U | U | C |
| Koivuranta, 1999 | U | U | U | U | C | U | C | C | C | U | C | C | U | C | U |
| McCall, 1999 | P | U | U | U | P | U | P | U | U | C | C | P | C | C | U |
| Sinha, 1999 | U | U | U | P | P | U | U | U | P | U | C | C | U | C | U |
| Diemunsch, 1998 | P | U | U | U | C | U | U | U | C | P | C | U | U | C | C |
| Hamid, 1998 | U | U | U | U | U | U | U | U | U | U | U | P | U | U | U |
| Tramer, 1998 | C | U | U | U | C | NA | C | C | P | C | C | C | U | C | C |

| | | | | | | | | | | | | | | | |
|---|---|---|---|---|---|---|---|---|---|---|---|---|---|---|---|
| Diemunsch, 1997b | P | U | U | U | C | U | U | U | C | P | C | U | C | C | C |
| Diemunsch, 1997c | P | U | U | U | P | U | U | U | U | C | C | U | C | C | C |
| Graczyk, 1997 | P | U | U | U | C | U | U | U | C | P | C | U | U | C | C |
| Klockgether-Radke, 1997 | C | U | U | U | P | U | U | U | P | P | C | U | U | C | C |
| Koivuranta, 1997 | P | U | U | P | C | U | U | U | C | U | C | C | C | C | P |
| Korttila, 1997 | P | U | U | U | C | NA | U | U | C | P | C | C | C | C | P |
| Kovac, 1997 | P | U | U | U | C | P | P | U | C | P | C | U | C | C | C |
| Mikawa, 1997 | P | U | U | U | C | NA | P | U | C | P | C | P | U | C | C |
| Monagle, 1997 | U | U | U | U | P | P | P | P | P | P | U | P | U | U | C |
| Morton, 1997 | U | U | U | U | U | U | U | U | C | U | C | C | U | C | C |
| Patel, 1997 | P | U | U | U | C | C | C | P | C | U | C | C | U | C | U |
| Purhonen, 1997 | C | U | U | U | P | U | U | U | P | P | U | C | U | C | P |
| Scuderi, 1997 | P | U | U | U | C | U | U | U | C | P | U | U | U | C | P |
| Taylor, 1997 | U | U | U | C | P | P | C | P | P | U | C | P | U | C | U |
| Ulusoy, 1997 | P | U | U | U | P | U | U | U | C | U | C | U | U | C | P |
| Warriner, 1997 | U | U | U | C | C | U | U | U | C | P | C | U | U | C | C |
| Capouet, 1996 | U | U | U | U | P | P | C | P | C | U | C | P | U | C | U |
| Kovac, 1996 (CR: Pearman 1994) | P | U | U | U | P | P | C | P | C | P | C | C | U | C | C |
| Scholz, 1996 | U | U | U | U | U | U | U | U | C | P | C | U | U | C | P |
| Desilva, 1995 | P | U | U | U | P | U | P | U | U | U | C | C | U | C | C |
| Litman, 1995 | U | U | U | U | C | U | U | U | C | U | U | U | U | P | C |
| Paech, 1995 | P | U | U | U | C | NA | P | U | C | C | C | P | U | C | C |
| Paxton, 1995a | U | U | U | U | P | U | P | U | C | P | U | U | U | U | P |
| Kaufmann, 1994 | P | U | U | U | P | U | C | C | C | P | C | C | U | C | C |
| Ummenhofer, 1994 | U | U | U | U | C | U | P | U | C | P | U | U | U | C | P |
| Dupeyron, 1993 | U | U | U | U | C | C | P | P | U | U | C | U | U | C | U |
| Helmers, 1993 | P | U | U | C | C | C | P | U | C | P | C | P | U | C | U |
| Raphael, 1993 | U | U | U | U | C | C | U | U | C | P | C | C | P | C | P |
| Du Pen, 1992 | P | U | U | U | NA | C | P | U | P | U | C | U | U | C | U |
| Kovac, 1992 (CR: Mckenzie, 1993a) | P | U | U | U | P | U | P | U | C | P | C | U | C | C | C |
| ***Non-randomized clinical trials*** | | | | | | | | | | | | | | | |
| Charbit, 2005 | P | U | U | U | C | U | C | U | C | C | C | U | U | C | C |
| ***Controlled before-after studies*** | | | | | | | | | | | | | | | |
| Wagner, 2007 | U | U | U | U | P | U | U | U | C | U | C | U | U | C | U |

**Abbreviations:** C, complete; NA, not applicable; P, partial; U, unclear.

**Items:**

1. Were the harms pre-defined using standard or precise definitions?
2. Were serious events precisely defined?
3. Were severe events precisely defined?
4. Were the number of deaths in each study group specified or were the reason(s) for unclear specifying given?
5. Was the mode of harms collection specified as active?
6. Was the mode of harms collection specified as passive?
7. Did the study specify who collected the harms?
8. Did the study specify the training or background of who ascertained the harms?
9. Did the study specify the timing and frequency of collection of the harms
10. Did the authors use standard scales or checklists for harms collection?
11. Did the authors specify if the harms reported encompass all the events collected or a selected sample?
12. Was the overall number of participants that withdrew or were lost to follow-up, including each reason why, specified for each study group?
13. Was the number of participants who withdrew due to harms specified for each study group?
14. Did the author(s) specify the number and type of harmful events for each study group?
15. Did the author(s) specify the type of analyses undertaken for harms data?

**Appendix 8. Aggregate McHarm appraisal results**

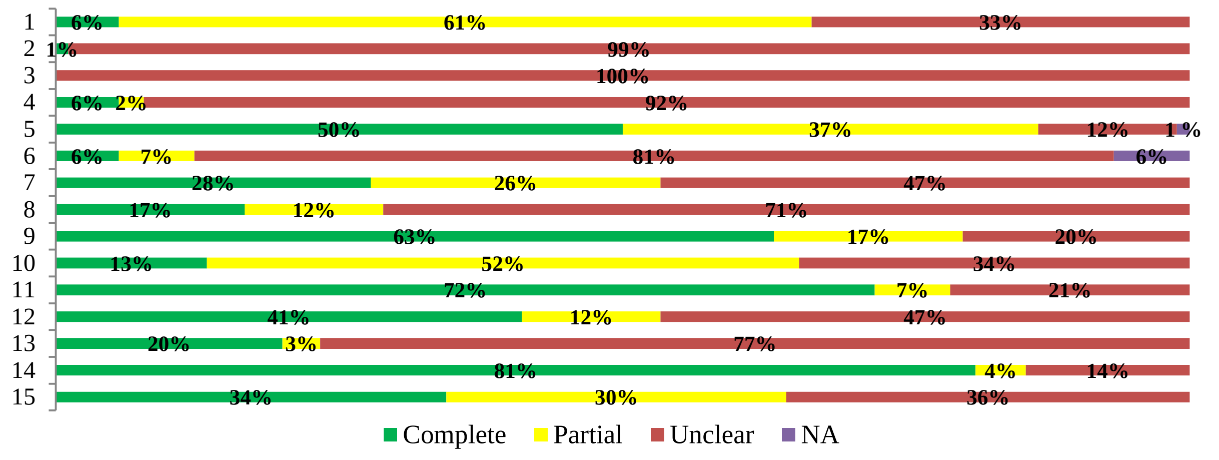


**Items:**

1. Were the harms pre-defined using standard or precise definitions?
2. Were serious events precisely defined?
3. Were severe events precisely defined?
4. Were the number of deaths in each study group specified or were the reason(s) for unclear specifying given?
5. Was the mode of harms collection specified as active?
6. Was the mode of harms collection specified as passive?
7. Did the study specify who collected the harms?
8. Did the study specify the training or background of who ascertained the harms?
9. Did the study specify the timing and frequency of collection of the harms
10. Did the authors use standard scales or checklists for harms collection?
11. Did the authors specify if the harms reported encompass all the events collected or a selected sample?
12. Was the overall number of participants that withdrew or were lost to follow-up, including each reason why, specified for each study group?
13. Was the number of participants who withdrew due to harms specified for each study group?
14. Did the author(s) specify the number and type of harmful events for each study group?
15. Did the author(s) specify the type of analyses undertaken for harms data?

**Appendix 9. Comparison adjusted funnel plots**

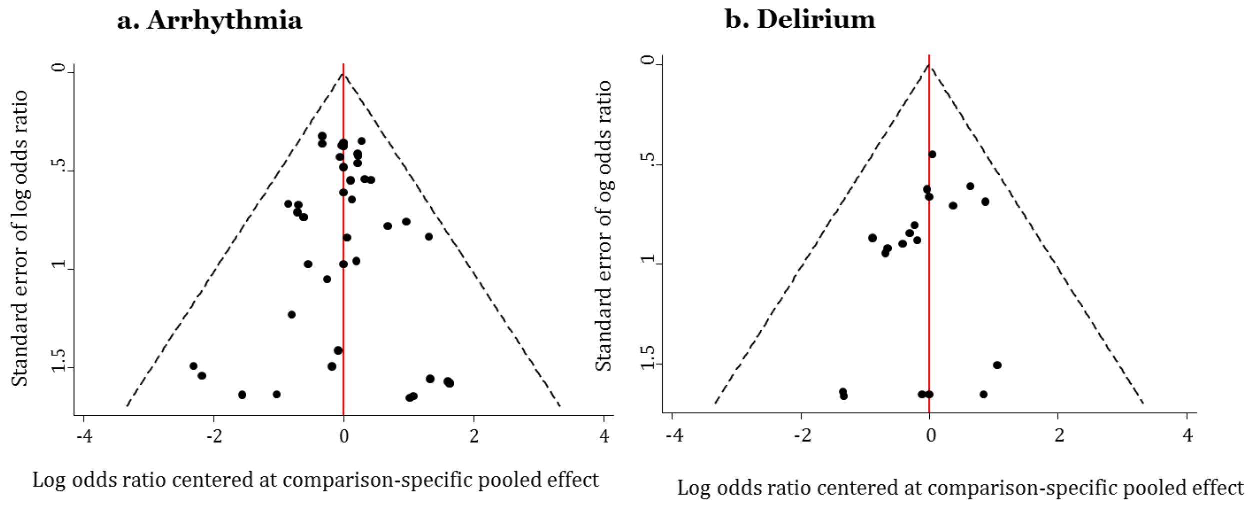

## Appendix 10. Definitions of arrhythmia

| Study | Definition of arrhythmia |
|---|---|
| Sahoo, 2012 | No definition for bradycardia reported |
| El-Deeb, 2011b | No definition for bradycardia reported |
| Choi, 2010 | 'Episodes of arrhythmias (i.e., atrial fibrillation or flutter, paroxysmal supraventricular tachycardia, atrial or ventricular premature contraction, or ventricular tachycardia) observed via ECG during the ICU stay were also recorded'; No specific definitions provided |
| Ekinci, 2009 | No definition for tachycardia reported |
| Raid, 2009 | No definition for OCR requiring atropine reported |
| Owczuk, 2008 | No definition for bradycardia requiring atropine reported |
| Kelsaka, 2006 | No definition for bradycardia reported |
| Gan, 2005 | No definition for bradycardia reported |
| Kahlil, 2005 | No definition for 'tachycardia & nodal arrhythmia' reported |
| Treschan, 2005 | No definition for OCR reported |
| Binstock, 2004 | No definition for tachycardia reported |
| Eberhart, 2004 | No definition for bradycardia (OCR) requiring atropine reported |
| Hanaoka, 2004 | No definition for ventricular fibrillation (cardiac arrest) reported |
| Samarkandi, 2004 | No definition for OCR requiring atropine reported |
| Philip, 2000 | No definition for 'sinus arrhythmia' reported |
| Subramaniam, 2000 | No definition for OCR requiring atropine reported |
| Tramer, 1998 | 'A significant OCR was defined as an acute decrease in heart rate of 20% or greater associated with traction on an eye muscle' |
| Diemunsch, 1997b | No definition for bradycardia reported |
| Klockgether-Radke, 1997 | No definition for 'OCR' reported |
| Korttila, 1997 | No definition for bradycardia reported |
| Morton, 1997 | No definition for bradycardia reported |
| Purhonen, 1997 | Bradycardia defined as 'heart rate <50 bpm' |
| Capouet, 1996 | No definition for 'bradycarida treated with an anticholinergic' reported |
| Hennes, 1996 | No definition for bradycardia reported |
| Paxton, 1995a | No definition of nodal rhythms reported |
| Helmers, 1993 | No definition for bradycardia reported |

**Abbreviations**: Bpm, beats per minute; ECG, electrocardiogram; ICU, intensive care unit; OCR, oculocardiac reflex.

## Appendix 11. Network meta-analysis for all outcomes for all time periods

| ***Treatment Comparison*** | ***# of studies*** | ***MA estimate: OR (95% CI)*** | ***NMA estimate: OR (95% CI)*** | ***# of studie s*** | ***MA estimate: OR (95% CI)*** | ***NMA estimate: OR (95% CI)*** |
|---|---|---|---|---|---|---|
| | **Arrhythmia (all ages) - 31 RCTs and 6,623 patients** | | | **Arrhythmia (children) - 9 RCTs and 1,572 patients** | | |
| Ondansetron vs Placebo | 18 | 0.93 (0.70–1.23) | 0.92 (0.69–1.21) | 8 | 1.12 (0.54–2.33) | 1.09 (0.56–2.14) |
| Granisetron vs Placebo | 2 | 1.12 (0.40–3.11) | 1.12 (0.40–3.11) | NA | NA | NA |
| Dolasetron vs Placebo | 7 | 0.67 (0.45–1.00) | 0.68 (0.46–1.01) | NA | NA | NA |
| Tropisetron vs Placebo | 4 | 0.93 (0.48–1.79) | 0.91 (0.49–1.68) | NA | NA | NA |
| Ondansetron+DEX vs Placebo | 2 | 0.78 (0.19–3.14) | 0.52 (0.16–1.68) | 2 | 0.78 (0.19–3.14) | 0.72 (0.20–2.66) |
| Ramosetron vs Placebo | 1 | 1.36 (0.66–2.80) | 1.15 (0.61–2.15) | NA | NA | NA |
| Granisetron+DEX vs Placebo | 2 | 2.63 (0.75–9.29) | 2.96 (1.11–7.94) | 1 | 4.89 (1.15–20.79) | 5.15 (1.33–19.91) |
| Dolasetron+DROP vs Placebo | 1 | 0.91 (0.45–1.84) | 0.91 (0.48–1.73) | NA | NA | NA |
| Granisetron vs Ondansetron | NA | NA | 1.22 (0.43–3.53) | NA | NA | NA |
| Dolasetron vs Ondansetron | 1 | 0.73 (0.28–1.87) | 0.74 (0.46–1.18) | NA | NA | NA |
| Tropisetron vs Ondansetron | 1 | 1.10 (0.33–3.65) | 0.99 (0.52–1.90) | NA | NA | NA |
| Ondansetron+DEX vs Ondansetron | 1 | 0.63 (0.09–4.24) | 0.56 (0.17–1.86) | 1 | 0.63 (0.09–4.24) | 0.66 (0.17–2.59) |
| Ramosetron vs Ondansetron | 1 | 1.07 (0.53–2.15) | 1.25 (0.67–2.34) | NA | NA | NA |
| Granisetron+DEX vs Ondansetron | NA | NA | 3.23 (1.17–8.95) | NA | NA | 4.71 (1.08–20.46) |
| Dolasetron+DROP vs Ondansetron | NA | NA | 0.99 (0.49–1.99) | NA | NA | NA |
| Dolasetron vs Granisetron | NA | NA | 0.61 (0.20–1.81) | NA | NA | NA |
| Tropisetron vs Granisetron | NA | NA | 0.81 (0.25–2.66) | NA | NA | NA |
| Ondansetron+DEX vs Granisetron | NA | NA | 0.46 (0.10–2.19) | NA | NA | NA |
| Ramosetron vs Granisetron | NA | NA | 1.02 (0.31–3.39) | NA | NA | NA |
| Granisetron+DEX vs Granisetron | NA | NA | 2.64 (0.64–10.92) | NA | NA | NA |
| Dolasetron+DROP vs Granisetron | NA | NA | 0.81 (0.24–2.70) | NA | NA | NA |
| Tropisetron vs Dolasetron | NA | NA | 1.34 (0.64–2.77) | NA | NA | NA |
| Ondansetron+DEX vs Dolasetron | NA | NA | 0.76 (0.22–2.64) | NA | NA | NA |
| Ramosetron vs Dolasetron | NA | NA | 1.69 (0.81–3.53) | NA | NA | NA |
| Granisetron+DEX vs Dolasetron | NA | NA | 4.37 (1.51–12.62) | NA | NA | NA |
| Dolasetron+DROP vs Dolasetron | 1 | 1.33 (0.63–2.77) | 1.34 (0.69–2.58) | NA | NA | NA |
| Ondansetron+DEX vs Tropisetron | NA | NA | 0.57 (0.15–2.15) | NA | NA | NA |
| Ramosetron vs Tropisetron | NA | NA | 1.26 (0.53–3.01) | NA | NA | NA |
| Granisetron+DEX vs Tropisetron | NA | NA | 3.27 (1.02–10.43) | NA | NA | NA |
| Dolasetron+DROP vs Tropisetron | NA | NA | 1.00 (0.41–2.43) | NA | NA | NA |
| Ramosetron vs Ondansetron+DEX | NA | NA | 2.22 (0.59–8.42) | NA | NA | NA |
| Granisetron+DEX vs Ondansetron+DEX | 2 | 8.10 (1.92–34.13) | 5.75 (1.71–19.34) | 1 | 7.67 (1.47–40) | 7.12 (1.66–30.63) |
| Dolasetron+DROP vs Ondansetron+DEX | NA | NA | 1.76 (0.46–6.77) | NA | NA | NA |

| | | | | | | |
|---|---|---|---|---|---|---|
| Granisetron+DEX vs Ramosetron | NA | NA | 2.59 (0.81–8.31) | NA | NA | NA |
| Dolasetron+DROP vs Ramosetron | NA | NA | 0.79 (0.32–1.95) | NA | NA | NA |
| Dolasetron+DROP vs Granisetron+DEX | NA | NA | 0.31 (0.09–0.99) | NA | NA | NA |
| **Between-study heterogeneity** | **0.01** | | | **0.02** | | |
| **Design-by-treatment interaction model for inconsistency ($\chi^2$, d.f., P-value, heterogeneity)** | **3.49 (10, 0.968, 0.01)** | | | **0.13 (2, 0.938, 0.32)** | | |
| | **Delirium (all ages) - 18 RCTs and 3,652 patients** | | | **Delirium (children) - 4 RCTs and 1,220 patients** | | |
| Ondansetron vs Placebo | 11 | 0.84 (0.53–1.32) | 0.83 (0.53–1.31) | 2 | 0.83 (0.19–3.60) | 0.77 (0.23–2.61) |
| Granisetron vs Placebo | 3 | 0.29 (0.08–1.08) | 0.30 (0.08–1.12) | NA | NA | NA |
| Dolasetron vs Placebo | 1 | 3.34 (0.13–85.47) | 3.34 (0.13–85.52) | NA | NA | NA |
| Tropisetron vs Placebo | 3 | 1.45 (0.58–3.66) | 1.45 (0.58–3.66) | 2 | 1.35 (0.45–4.01) | 1.38 (0.39–4.8) |
| Dolasetron+DEX vs Placebo | NA | NA | 3.54 (0.11–116.45) | NA | NA | NA |
| Granisetron vs Ondansetron | NA | NA | 0.36 (0.09–1.43) | NA | NA | NA |
| Dolasetron vs Ondansetron | NA | NA | 4.00 (0.15–105.86) | NA | NA | NA |
| Tropisetron vs Ondansetron | NA | NA | 1.74 (0.62–4.88) | NA | NA | 1.78 (0.32–9.9) |
| Dolasetron+DEX vs Ondansetron | NA | NA | 4.24 (0.13–143.81) | NA | NA | NA |
| Dolasetron vs Granisetron | NA | NA | 11.13 (0.34–368.08) | NA | NA | NA |
| Tropisetron vs Granisetron | NA | NA | 4.85 (0.97–24.11) | NA | NA | NA |
| Dolasetron+DEX vs Granisetron | NA | NA | 11.80 (0.28–492.67) | NA | NA | NA |
| Tropisetron vs Dolasetron | NA | NA | 0.44 (0.01–12.68) | NA | NA | NA |
| Dolasetron+DEX vs Dolasetron | 1 | 1.06 (0.29–3.88) | 1.06 (0.29–3.88) | NA | NA | NA |
| Dolasetron+DEX vs Tropisetron | NA | NA | 2.43 (0.07–90.26) | NA | NA | NA |
| **Between-study heterogeneity** | **0.00** | | | **0.18** | | |
| **Design-by-treatment interaction model for inconsistency ($\chi^2$, d.f., P-value, heterogeneity)** | **0.31 (2, 0.857, 0.00)** | | | **NA** | | |

**Abbreviations:** DEX dexamethasone, DROP droperidol (intravenous), METO Metoclopramide (intravenous), PONV post-operative nausea and vomiting.

**Appendix 12. Plot of the surface under the cumulative ranking (SUCRA) curves for the 9 treatments included in Arrhythmia.**

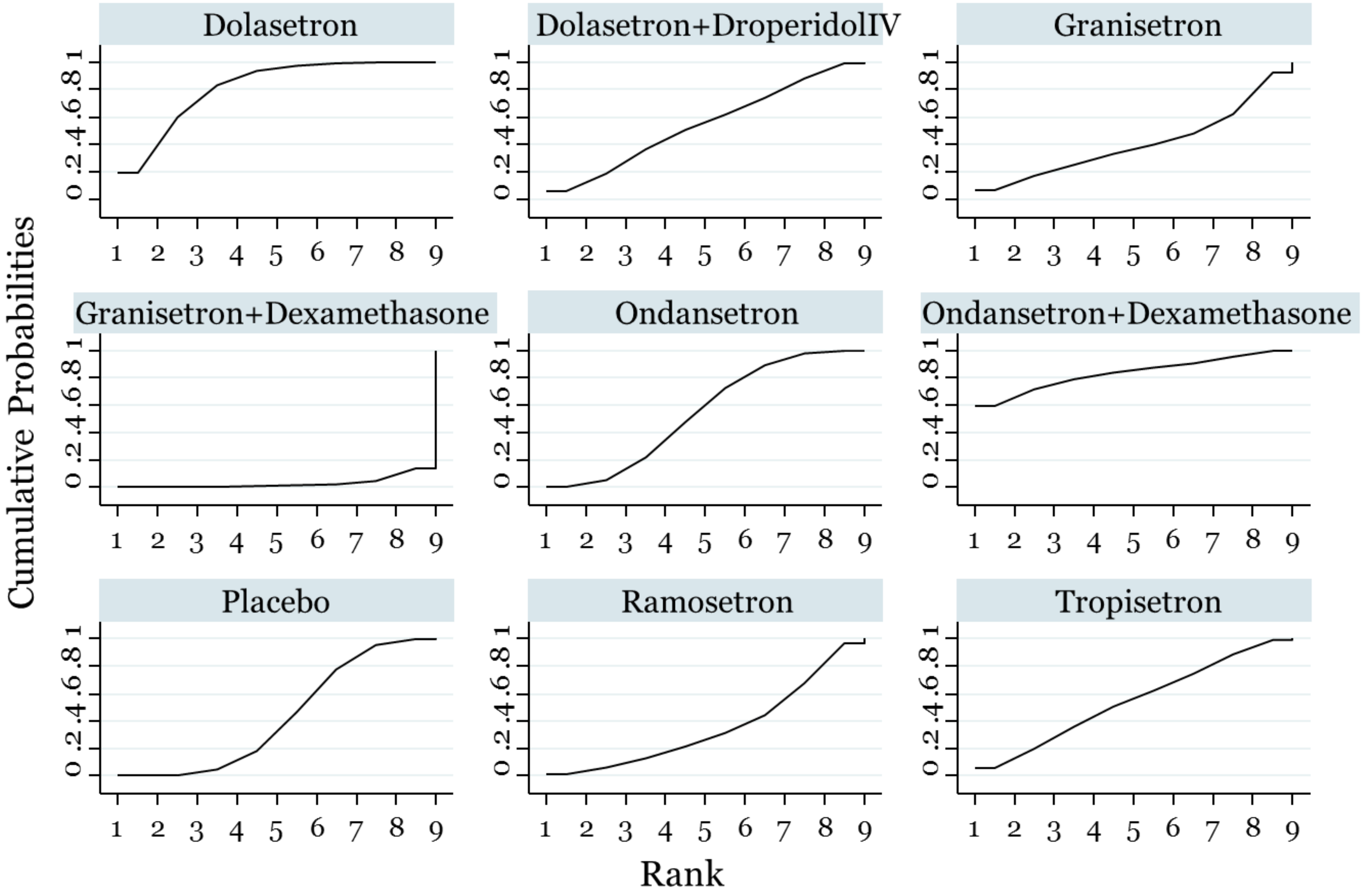

## Appendix 13: Subgroup analysis for Arrhythmia and Delirium

| | Intra surgery - RCTs only | | | All time periods - RCTs only - sensitivity analysis for Risk of Bias | | | All time periods - all study designs | | |
|---|---|---|---|---|---|---|---|---|---|
| ***Treatment Comparison*** | ***# of studies*** | ***MA estimate: OR (95% CI)*** | ***NMA estimate: OR (95% CI)*** | ***# of studies*** | ***MA estimate: OR (95% CI)*** | ***NMA estimate: OR (95% CI)*** | ***# of studies*** | ***MA estimate: OR (95% CI)*** | ***NMA estimate: OR (95% CI)*** |
| | **Arrhythmia** | | | | | | | | |
| Ondansetron vs Placebo | 16 | 0.89 (0.65-1.23) | 0.87 (0.64-1.2) | 18 | 0.93 (0.7-1.23) | 0.92 (0.69-1.21) | | | |
| Granisetron vs Placebo | 1 | 3.12 (0.13-77.52) | 3.12 (0.13-77.37) | 1 | 3.12 (0.13-77.52) | 3.12 (0.13-77.36) | | | |
| Dolasetron vs Placebo | 4 | 0.56 (0.34-0.92) | 0.57 (0.35-0.93) | 7 | 0.67 (0.45-1) | 0.68 (0.46-1.01) | | | |
| Tropisetron vs Placebo | 4 | 0.93 (0.48-1.79) | 0.9 (0.49-1.66) | 4 | 0.93 (0.48-1.79) | 0.91 (0.49-1.68) | | | |
| Ondansetron+Dexamethasone vs Placebo | 2 | 0.78 (0.19-3.14) | 0.51 (0.16-1.66) | 2 | 0.78 (0.19-3.14) | 0.52 (0.16-1.68) | | | |
| Ramosetron vs Placebo | 1 | 1.48 (0.73-3) | 1.48 (0.73-3) | 1 | 1.36 (0.66-2.8) | 1.15 (0.61-2.15) | | | |
| Granisetron+Dexamethasone vs Placebo | 2 | 2.63 (0.75-9.29) | 2.95 (1.1-7.91) | 2 | 2.63 (0.75-9.29) | 2.96 (1.11-7.94) | | | |
| Dolasetron+DroperidolIV vs Placebo | 1 | 0.91 (0.45-1.84) | 0.84 (0.44-1.62) | 1 | 0.91 (0.45-1.84) | 0.91 (0.48-1.73) | | | |
| Granisetron vs Ondansetron | NA | NA | 3.56 (0.14-89.88) | NA | NA | 3.4 (0.14-85.46) | | | |
| Dolasetron vs Ondansetron | 1 | 0.73 (0.28-1.87) | 0.66 (0.38-1.14) | 1 | 0.73 (0.28-1.87) | 0.74 (0.46-1.18) | | | |
| Tropisetron vs Ondansetron | 1 | 1.1 (0.33-3.65) | 1.03 (0.53-1.99) | 1 | 1.1 (0.33-3.65) | 0.99 (0.52-1.9) | | | |
| Ondansetron+Dexamethasone vs Ondansetron | 1 | 0.63 (0.09-4.24) | 0.58 (0.17-1.94) | 1 | 0.63 (0.09-4.24) | 0.56 (0.17-1.86) | | | |
| Ramosetron vs Ondansetron | NA | NA | 1.69 (0.78-3.67) | 1 | 1.07 (0.53-2.15) | 1.25 (0.67-2.34) | | | |
| Granisetron+Dexamethasone vs Ondansetron | NA | NA | 3.38 (1.21-9.42) | NA | NA | 3.23 (1.17-8.95) | | | |
| Dolasetron+DroperidolIV vs Ondansetron | NA | NA | 0.96 (0.47-1.98) | NA | NA | 0.99 (0.49-1.99) | | | |
| Dolasetron vs Granisetron | NA | NA | 0.18 (0.01-4.75) | NA | NA | 0.22 (0.01-5.54) | | | |
| Tropisetron vs Granisetron | NA | NA | 0.29 (0.01-7.59) | NA | NA | 0.29 (0.01-7.66) | | | |
| Ondansetron+Dexamethasone vs Granisetron | NA | NA | 0.16 (0.01-5.01) | NA | NA | 0.17 (0.01-5.07) | | | |
| Ramosetron vs Granisetron | NA | NA | 0.47 (0.02-12.72) | NA | NA | 0.37 (0.01-9.7) | | | |
| Granisetron+Dexamethasone vs Granisetron | NA | NA | 0.95 (0.03-27.27) | NA | NA | 0.95 (0.03-27.38) | | | |
| Dolasetron+DroperidolIV vs Granisetron | NA | NA | 0.27 (0.01-7.17) | NA | NA | 0.29 (0.01-7.7) | | | |
| Tropisetron vs Dolasetron | NA | NA | 1.56 (0.72-3.4) | NA | NA | 1.34 (0.64-2.77) | | | |
| Ondansetron+Dexamethasone vs Dolasetron | NA | NA | 0.88 (0.25-3.17) | NA | NA | 0.76 (0.22-2.64) | | | |
| Ramosetron vs Dolasetron | NA | NA | 2.57 (1.09-6.06) | NA | NA | 1.69 (0.81-3.53) | | | |
| Granisetron+Dexamethasone vs Dolasetron | NA | NA | 5.13 (1.72-15.37) | NA | NA | 4.37 (1.51-12.62) | | | |
| Dolasetron+DroperidolIV vs Dolasetron | 1 | 1.33 (0.63-2.77) | 1.46 (0.75-2.87) | NA | 1.33 (0.63-2.77) | 1.34 (0.69-2.58) | | | |
| Ondansetron+Dexamethasone vs Tropisetron | NA | NA | 0.57 (0.15-2.14) | NA | NA | 0.57 (0.15-2.15) | | | |
| Ramosetron vs Tropisetron | NA | NA | 1.64 (0.64-4.21) | NA | NA | 1.26 (0.53-3.01) | | | |
| Granisetron+Dexamethasone vs Tropisetron | NA | NA | 3.29 (1.03-10.49) | NA | NA | 3.27 (1.02-10.43) | | | |
| Dolasetron+DroperidolIV vs Tropisetron | NA | NA | 0.94 (0.38-2.3) | NA | NA | 1 (0.41-2.43) | | | |
| Ramosetron vs | NA | NA | 2.9 (0.73-11.55) | NA | NA | 2.22 (0.59-8.42) | | | |

| | | | | | | | | | |
|---|---|---|---|---|---|---|---|---|---|
| Ondansetron+Dexamethasone | | | | | | | | | |
| Granisetron+Dexamethasone vs Ondansetron+Dexamethasone | 2 | 8.1 (1.92-34.13) | 5.8 (1.73-19.51) | 2 | 8.1 (1.92-34.13) | 5.75 (1.71-19.34) | | | |
| Dolasetron+DroperidolIV vs Ondansetron+Dexamethasone | NA | NA | 1.66 (0.43-6.4) | NA | NA | 1.76 (0.46-6.77) | | | |
| Granisetron+Dexamethasone vs Ramosetron | NA | NA | 2 (0.59-6.73) | NA | NA | 2.59 (0.81-8.31) | | | |
| Dolasetron+DroperidolIV vs Ramosetron | NA | NA | 0.57 (0.22-1.5) | NA | NA | 0.79 (0.32-1.95) | | | |
| Dolasetron+DroperidolIV vs Granisetron+Dexamethasone | NA | NA | 0.29 (0.09-0.93) | NA | NA | 0.31 (0.09-0.99) | | | |
| **Between-study heterogeneity** | | | **0.00** | | | **0.00** | | | |
| **Design-by-treatment interaction model for inconsistency ($\chi^2$, d.f., P-value, heterogeneity)** | | | **2.88 (9, 0.969, 0.04)** | | | **3.46 (10, 0.968, 0.01)** | | | |
| | **Delirium** | | | | | | | | |
| Ondansetron vs Placebo | 10 | 0.80 (0.50-1.28) | 0.80 (0.5-1.27) | 10 | 0.85 (0.54-1.35) | 0.85 (0.54-1.35) | 12 | 0.85 (0.54-1.34) | 0.85 (0.54-1.34) |
| Granisetron vs Placebo | 2 | 0.17 (0.02-1.51) | 0.19 (0.02-1.64) | 1 | 0.1 (0.01-1.92) | 0.1 (0.01-1.92) | 3 | 0.29 (0.08-1.08) | 0.3 (0.08-1.12) |
| Dolasetron vs Placebo | 1 | 3.34 (0.13-85.47) | 3.34 (0.13-85.56) | 1 | 3.34 (0.13-85.47) | 3.34 (0.13-85.54) | 1 | 3.34 (0.13-85.47) | 3.34 (0.13-85.54) |
| Tropisetron vs Placebo | 2 | 1.35 (0.45-4.01) | 1.35 (0.45-4.01) | 3 | 1.45 (0.58-3.66) | 1.45 (0.58-3.66) | 3 | 1.45 (0.58-3.66) | 1.45 (0.58-3.66) |
| Dolasetron+DEX vs Placebo | NA | NA | 0.24 (0.03-2.15) | NA | NA | 3.54 (0.11-116.48) | NA | NA | 3.54 (0.11-116.46) |
| Granisetron vs Ondansetron | NA | NA | 4.2 (0.16-111.21) | NA | NA | 0.12 (0.01-2.35) | NA | NA | 0.35 (0.09-1.4) |
| Dolasetron vs Ondansetron | NA | NA | 1.7 (0.52-5.56) | NA | NA | 3.93 (0.15-103.99) | NA | NA | 3.91 (0.15-103.41) |
| Tropisetron vs Ondansetron | NA | NA | 17.46 (0.36-855.21) | NA | NA | 1.71 (0.61-4.8) | NA | NA | 1.7 (0.61-4.76) |
| Dolasetron+DEX vs Ondansetron | NA | NA | 7.05 (0.63-78.61) | NA | NA | 4.17 (0.12-141.28) | NA | NA | 4.15 (0.12-140.47) |
| Dolasetron vs Granisetron | NA | NA | 0.4 (0.01-12.37) | NA | NA | 33.35 (0.41-2683.9) | NA | NA | 11.12 (0.34-367.78) |
| Tropisetron vs Granisetron | NA | NA | NA | NA | NA | 14.52 (0.66-321.2) | NA | NA | 4.84 (0.97-24.09) |
| Dolasetron+DEX vs Granisetron | NA | NA | NA | NA | NA | 35.35 (0.36-3434.22) | NA | NA | 11.79 (0.28-492.21) |
| Tropisetron vs Dolasetron | NA | NA | NA | NA | NA | 0.44 (0.01-12.68) | NA | NA | 0.44 (0.01-12.69) |
| Dolasetron+DEX vs Dolasetron | NA | NA | NA | 1 | 1.06 (0.29-3.88) | 1.06 (0.29-3.88) | 1 | 1.06 (0.29-3.88) | 1.06 (0.29-3.88) |
| Dolasetron+DEX vs Tropisetron | NA | NA | NA | NA | NA | 2.43 (0.07-90.28) | NA | NA | 2.43 (0.07-90.26) |
| **Between-study heterogeneity** | | | **0.00** | | | **0.00** | | | **0.00** |
| **Design-by-treatment interaction model for inconsistency ($\chi^2$, d.f., P-value,** | | | **0.63 (2, 0.729, 0.00)** | | | **NA** | | | **0.32 (2, 0.851, 0.00)** |

| | | | | | | | | | |
|---|---|---|---|---|---|---|---|---|---|
| **heterogeneity)** | | | | | | | | | |

**Appendix 14. Network meta-analysis results for Delirium.**

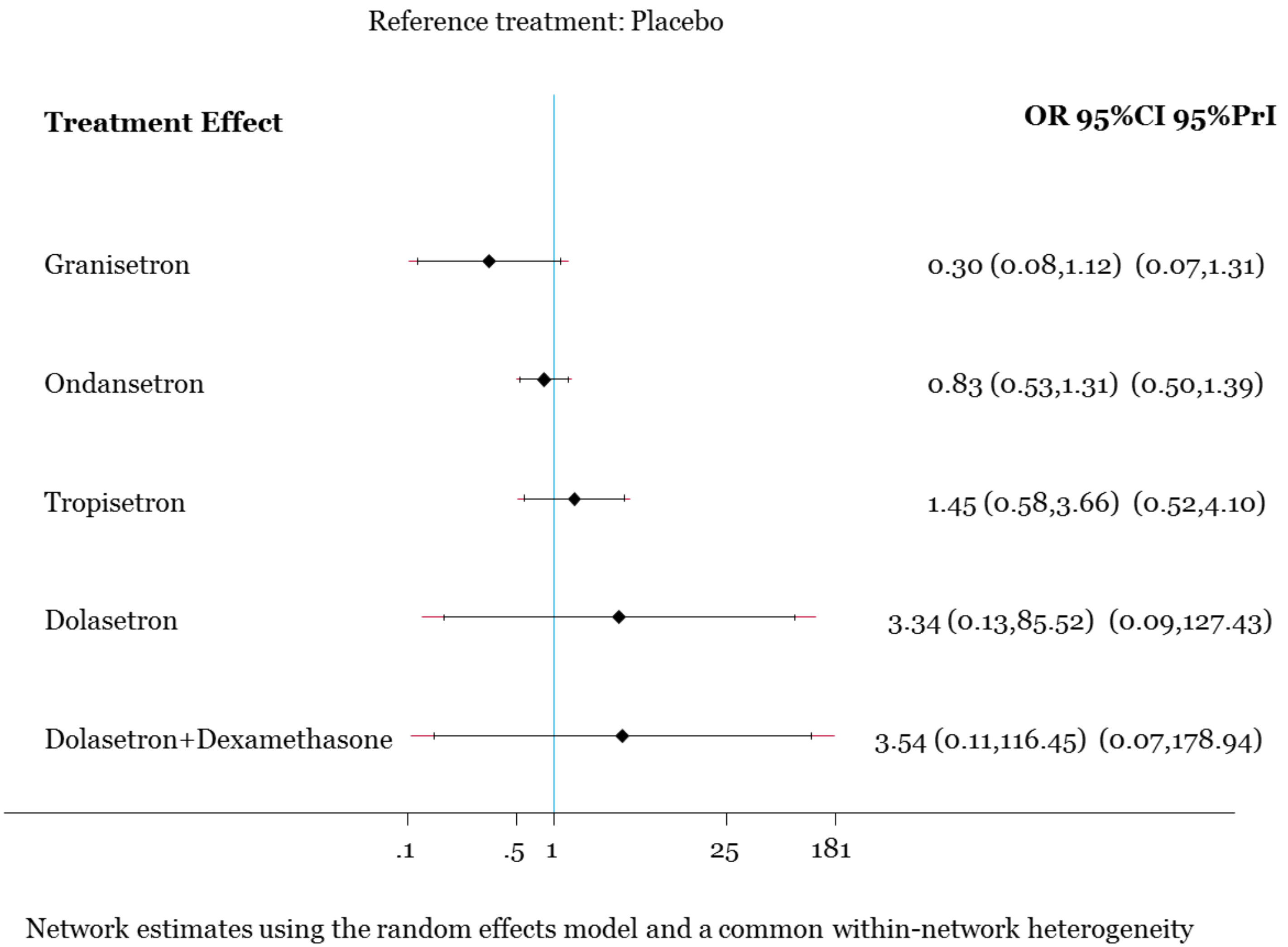


All treatments are compared to placebo. The black horizontal lines represent the 95% confidence intervals (CI) of the summary treatment effects and red horizontal lines the 95% predictive intervals (PrI). The results are presented on the odds ratio scale.

## Appendix 15. Definitions of delirium

| Study | Definition of delirium |
|---|---|
| Jain, 2009 | Confusion unspecified |
| Piper, 2008 | Restlessness, agitation unspecified |
| Sagir, 2007 | Agitation unspecified |
| Wagner, 2007 | Emergence delirium unspecified |
| Tosun, 2006 | Agitation unspecified |
| Khalil, 2005 | Agitation and aggressive behavior unspecified |
| Kathirvel, 2001 | Confusion/Dizziness unspecified |
| Jensen, 2000 | Restlessness unspecified |
| McCall, 1999 | Emergence was scored as 'where 1 = calm emergence, 2 = slight agitation or delirium, 3 = moderate agitation or delirium, and 4 = extreme agitation or delirium'; Delirium was considered a score of 3,4 |
| Sinha, 1999 | Confusion unspecified |
| Koivuranta, 1997 | Restlessness unspecified |
| Patel, 1997 | Anxiety/agitation unspecified |
| Kovac, 1996 (CR: Pearman, 1994c) | Anxiety/agitation unspecified |
| Paech, 1995 | Agitation unspecified |
| Kovac, 1992 (CR: Mckenzie, 1993a) | Anxiety/agitation unspecified |

**Appendix 16. Plot of the surface under the cumulative ranking (SUCRA) curves for the 6 treatments included in Delirium.**

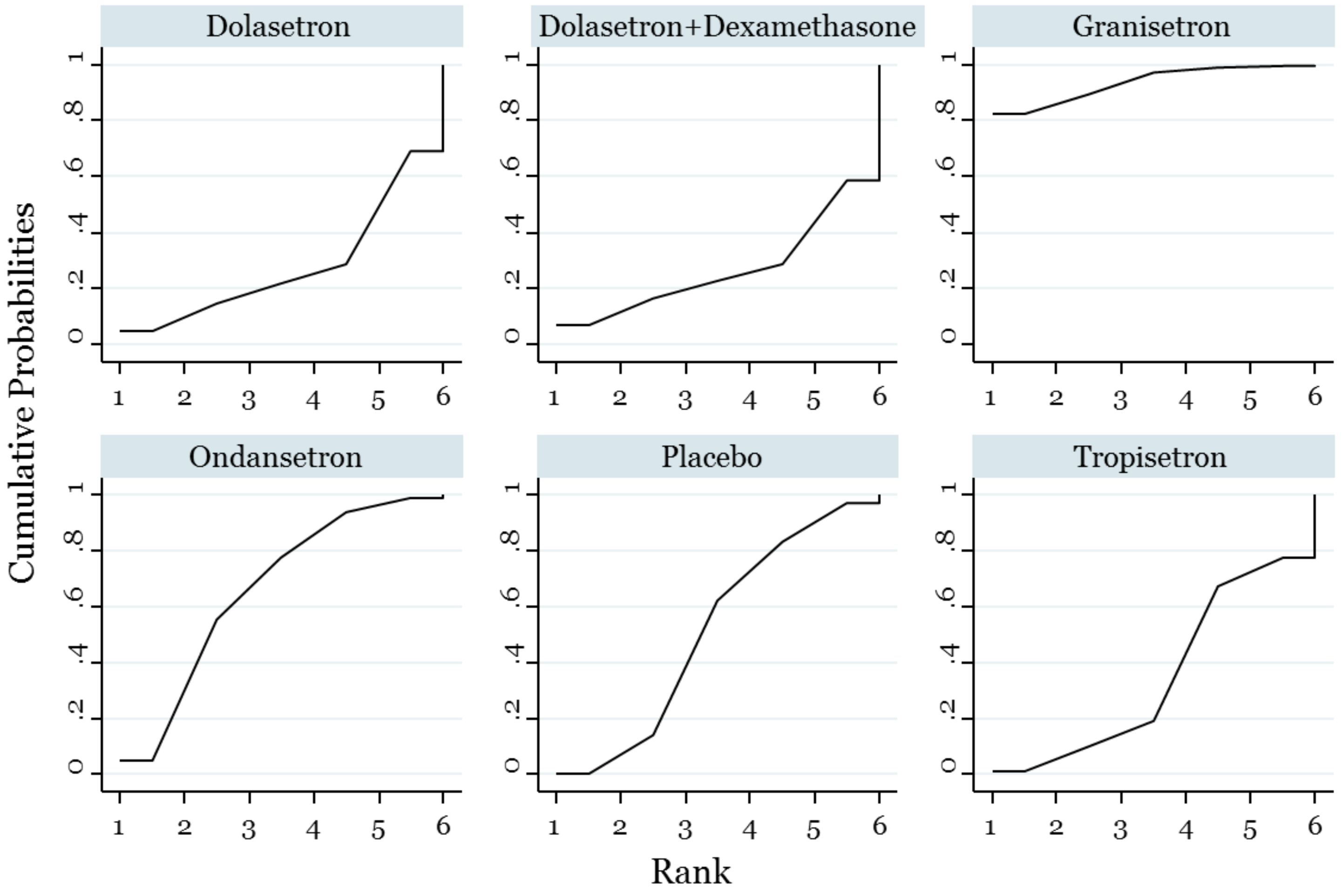

## Appendix 17. Included studies in our review versus previous systematic reviews

| Study | Tang, 2012 (NMA) | Carlisle, 2006 (Cochrane) | Rawlinson, 2012 | # of patients |
|---|---|---|---|---|
| ***Randomized clinical trials (n=112)*** | | | | |
| Choi, 2012 | NO | NO | NO | 120 |
| Wang, 2012 | NO | NO | NO | 130 |
| Ekinci, 2011 | NO | NO | NO | 100 |
| El-deeb, 2011b | NO | NO | NO | 160 |
| Gan, 2011 | NO | NO | NO | 619 |
| Gupta, 2011 | NO | NO | NO | 136 |
| Park, 2011 | NO | NO | NO | 90 |
| Ryu, 2011 | NO | NO | NO | 124 |
| Sahoo, 2011 | NO | NO | NO | 52 |
| Bilgin, 2010 | NO | NO | NO | 160 |
| Choi, 2010 | NO | NO | NO | 279 |
| Jee, 2010 | NO | NO | NO | 100 |
| Jokela, 2010 | NO | NO | NO | 80 |
| Mehta, 2010 | NO | NO | NO | 80 |
| Shakya, 2010 | NO | NO | NO | 80 |
| Singla, 2010 | NO | NO | NO | 702 |
| Feng, 2009 | NO | NO | NO | 192 |
| Jain, 2009 | NO | NO | NO | 87 |
| Riad, 2009 | NO | NO | NO | 100 |
| Rosow, 2009 | NO | NO | NO | 244 |
| Candiotti, 2008 | NO | NO | NO | 546 |
| Choi, 2008 | NO | NO | NO | 94 |
| Contreras-Dominguez, 2008 | YES | NO | NO | 150 |
| Kovac, 2008 | NO | NO | NO | 544 |
| Owczuk, 2008 | NO | NO | NO | 71 |
| Piper, 2008 | YES | NO | NO | 83 |
| Said-Ahmed, 2008 | NO | NO | NO | 50 |
| Bestas, 2007 | YES | NO | NO | 90 |
| Diemunsch, 2007 | NO | NO | NO | 892 |
| Gan, 2007 | NO | NO | NO | 766 |
| Han, 2007 | NO | NO | NO | 80 |
| Lee, 2007 | NO | NO | NO | 86 |
| Oksuz, 2007 | NO | NO | NO | 75 |
| Rusch, 2007 | NO | NO | NO | 228 |
| Sagir, 2007 | NO | NO | NO | 80 |
| Sandhu, 2007 | NO | NO | NO | 80 |
| Siddik-Sayyid, 2007 | NO | NO | NO | 129 |
| Bridge, 2006 | NO | NO | NO | 194 |
| Chan, 2006 | NO | NO | NO | 394 |
| Kelsaka, 2006 | NO | NO | NO | 50 |
| Sarvela, 2006 | NO | NO | NO | 87 |
| Tosun, 2006 | NO | YES | NO | 125 |

| | | | | |
|---|---|---|---|---|
| D'Angelo, 2005 | YES | NO | NO | 121 |
| Gan, 2005 | NO | NO | NO | 176 |
| Khalil, 2005 | NO | NO | NO | 670 |
| Kocamanoglu, 2005 | YES | NO | NO | 150 |
| Kontrimaviciute, 2005 | NO | NO | NO | 34 |
| Pirat, 2005 | YES | NO | NO | 150 |
| Treschan, 2005 | NO | NO | NO | 142 |
| White, 2005[†] | NO | NO | NO | 372 |
| Apfel, 2004 (CR: Jokela, 2009) | NO | YES | YES | 260 |
| Binstock, 2004 | NO | NO | NO | 101 |
| Eberhart, 2004 | NO | NO | NO | 304 |
| Hanaoka, 2004 | YES | NO | NO | 315 |
| Samakarndi, 2004 | NO | NO | NO | 80 |
| Charuluxananan, 2003 | NO | YES | NO | 180 |
| Loewen, 2003 | NO | YES | NO | 71 |
| O'Brien, 2003 | NO | YES | NO | 150 |
| Argiriadou, 2002 | NO | YES | NO | 87 |
| Gurkan, 2002 | NO | NO | NO | 150 |
| Dabbous, 2001 | NO | NO | NO | 173 |
| Kathirvel, 2001 | YES | YES | NO | 152 |
| Subramaniam, 2001 | NO | YES | NO | 135 |
| Ahmed, 2000 | NO | YES | NO | 139 |
| Charuluxananan, 2000 | NO | NO | NO | 80 |
| Jensen, 2000 | NO | YES | NO | 71 |
| Kreisler, 2000 | NO | YES | NO | 31 |
| Philip, 2000 | YES | YES | NO | 1030 |
| Zarate, 2000 | NO | YES | NO | 200 |
| Koivuranta, 1999 | NO | YES | NO | 88 |
| McCall, 1999 | NO | YES | NO | 100 |
| Sinha, 1999 | YES | YES | NO | 40 |
| Tsui, 1999 | NO | YES | NO | 121 |
| Diemunsch, 1998 | YES | YES | NO | 793 |
| Goodarzi, 1998 | NO | YES | NO | 81 |
| Hamid, 1998 | NO | YES | NO | 71 |
| Morris, 1998 | NO | YES | NO | 1044 |
| Scholz, 1998 | YES | YES | NO | 842 |
| Tramer, 1998 | NO | YES | NO | 157 |
| Diemunsch, 1997b | NO | YES | NO | 281 |
| Diemunsch, 1997c | NO | NO | NO | 337 |
| Graczyk, 1997 | NO | NO | NO | 635 |
| Klockgether-Radke, 1997 | NO | YES | NO | 120 |
| Koivuranta, 1997 | NO | YES | NO | 439 |
| Korttila, 1997 | YES | YES | NO | 517 |
| Kovac, 1997 | NO | NO | NO | 620 |
| Mikawa, 1997 | YES | YES | NO | 200 |
| Monagle, 1997 | NO | YES | NO | 91 |
| Morton, 1997 | NO | YES | NO | 427 |
| Patel, 1997 | NO | NO | NO | 429 |

| | | | | |
|---|---|---|---|---|
| Purhonen, 1997 | YES | YES | NO | 146 |
| Rung, 1997 | NO | NO | NO | 121 |
| Scuderi, 1997 | NO | YES | NO | 160 |
| Taylor, 1997 | NO | NO | NO | 523 |
| Ulusoy, 1997 | NO | NO | NO | 40 |
| Warriner, 1997 | YES | YES | NO | 374 |
| Ali-melkkila, 1996 | NO | NO | NO | 120 |
| Capouet, 1996 | YES | YES | NO | 385 |
| Kovac, 1996 (CR: Pearman, 1994c) | YES | YES | NO | 468 |
| Naguib, 1996 | YES | YES | NO | 132 |
| Rose, 1996a | NO | YES | NO | 136 |
| Desilva, 1995 | NO | NO | NO | 286 |
| Litman, 1995 | NO | YES | NO | 57 |
| Paech, 1995 | NO | YES | NO | 259 |
| Paxton, 1995a | NO | YES | NO | 60 |
| Kaufmann, 1994 | YES | YES | NO | 286 |
| Ummenhofer, 1994 | NO | YES | NO | 200 |
| Dupeyron, 1993 | NO | YES | NO | 243 |
| Helmers, 1993 | NO | YES | NO | 923 |
| Raphael, 1993 | NO | YES | NO | 123 |
| Du Pen, 1992 (CR: Scuderi, 1993) | NO | NO | NO | 500 |
| Kovac, 1992 (CRs: McKenzie, 1993a; Pearman, 1994a) | NO | NO | NO | 580 |
| ***Non randomized clinical trials (n=2)*** | | | | |
| Charbit, 2005 | NO | NO | NO | 85 |
| Lerman, 1996 | NO | YES | NO | 28 |
| ***Controlled before-after study (n=1)*** | | | | |
| Wagner, 2007 | NO | NO | NO | 66 |
| **Total included studies** | 66 | 217 | 4 | |

**Note:** †Unpublished data (White, 2005).

**Abbreviations:** CR, companion report; NMA, network meta-analysis; NO, study wasn't included in review; YES, study was included in review.
